# Transcriptome sequencing of a keystone aquatic herbivore yields insights on the temperature-dependent metabolism of essential lipids

**DOI:** 10.1186/s12864-019-6268-y

**Published:** 2019-11-21

**Authors:** Heidrun S. Windisch, Patrick Fink

**Affiliations:** 1 Heinrich-Heine-University, Institute for Cell Biology and Zoology, Universitätsstrasse 1, 40225 Düsseldorf, Germany; 2 Fraunhofer IME, Institute for Molecular Ecology, Am Aberg 1, 57392 Schmallenberg, Germany; 3 Institute for Zoology, University of Cologne, Zülpicher Strasse 47b, 50674 Köln, Germany; 4 Department River Ecology, Helmholtz Centre for Environmental Science, Brückstrasse 3a, 39114 Magdeburg, Germany; 5 Department Aquatic Ecosystem Analysis and Management, Helmholtz Centre for Environmental Science, Brückstrasse 3a, 39114 Magdeburg, Germany

**Keywords:** Omega-3 fatty acids, Eicosapentaenoic acid, Temperature, Gene expression, *Daphnia*

## Abstract

**Background:**

Nutritional quality of phytoplankton is a major determinant of the trophic transfer efficiency at the plant-herbivore interface in freshwater food webs. In particular, the phytoplankton’s content of the essential polyunsaturated omega-3 fatty acid eicosapentaenoic acid (EPA) has been repeatedly shown to limit secondary production in the major zooplankton herbivore genus *Daphnia*. Despite extensive research efforts on the biological model organism *Daphni*a, and the availability of several *Daphnia* genomes, little is known regarding the molecular mechanisms underlying the limitations in *Daphnia* related to dietary EPA availability.

**Results:**

We used RNA-seq to analyse the transcriptomic response of *Daphnia magna* which were fed with two different diets — each with or without supplementation of EPA — at two different temperature levels (15 and 20 °C). The transcripts were mapped to the *D. magna* genome assembly version 2.4, containing 26,646 translations. When *D. magna* fed on green alga, changing the temperature provoked a differential expression of 2001 transcripts, and in cyanobacteria-fed daphnia, 3385 transcripts were affected. The supplementation of EPA affected 1635 (on the green algal diet), or 175 transcripts (on the cyanobacterial diet), respectively. Combined effects for diet and temperature were also observed (669 for the green algal and 128 transcripts for the cyanobacterial diet). Searching for orthologous genes (COG-analysis) yielded a functional overview of the altered transcriptomes. Cross-matched transcript sets from both feed types were compiled to illuminate core responses to the factors temperature and EPA-supplementation.

**Conclusions:**

Our highly controlled eco-physiological experiments revealed an orchestrated response of genes involved in the transformation and signalling of essential fatty acids, including eicosanoid-signalling pathways with potential immune functions. We provide an overview of downstream-regulated genes, which contribute to enhance growth and reproductive output. We also identified numerous EPA-responsive candidate genes of yet unknown function, which constitute new targets for future studies on the molecular basis of EPA-dependent effects at the freshwater plant-herbivore interface.

## Background

Primary producer biomass is typically of poor quality in herbivores, which limits the trophic transfer of energy through food webs to higher trophic levels (trophic transfer efficiency) [1]. In aquatic environments, the photosynthetic base of the food web consists of small unicellular phytoplankton that is consumed by herbivorous zooplankton. Several constraints on algal food quality have been demonstrated, as algae can be hard to either ingest or digest by herbivores [2, 3]. Further, they can provide an unbalanced supply (stoichiometry) of nutrients [4]. In many cases their biochemical composition does not meet the herbivores’ demands in essential nutritional compounds, such as essential fatty acids [5, 6], sterols [7, 8], vitamins [9], or amino acids [10, 11].

In freshwater ecosystems, crustacean zooplankton of the genus *Daphnia* are the major pelagic herbivores, and form a crucial link between primary producers and consumers [12]. Beyond their key role in freshwater food webs, daphnids are a well-established model system of environmental toxicology, experimental ecology and evolution, due to their ecological importance and exceptionally high level of phenotypic plasticity [13–16]. The genomes of several *Daphnia* species have been sequenced, and it is therefore one of the few animal genera for which extensive ecological and genomic information is available [17, 18]. Interestingly, all the complex and plastic responses of daphnids are generated from a relatively small genome [17, 19]. This makes *Daphnia* an excellent animal model for gene expression studies in response to environmental cues, such as kairomone signalling of vertebrate and invertebrate predation, exposure to parasite spores, crowding, and grazing on toxic and non-toxic food sources [20–22].

Daphnids have been repeatedly shown to be highly affected by diets with an inappropriate supply of essential fatty acids, as they are unselective filter-feeders that cannot preferentially take up algal cells rich in particular lipids [23]. A lack or limiting availability of certain omega-3 (ω3, and to a lesser degree ω6) polyunsaturated fatty acids (PUFAs) has been shown to constrain somatic growth, reproduction, and population growth in several *Daphnia* species [24–26]. This is due to the fact that ω3 and ω6 PUFAs typically can only be synthesized by primary producers, and not by animals [5, 27–29]. These ‘families’ of PUFAs can therefore be considered as essential dietary constituents for most animals, including *Daphnia* [30].

Beyond their role in growth and reproduction, PUFAs are well recognised critical components of the so-called ‘homeoviscous adaptation’ of biological membranes to low temperatures [31]. This concept implies an incorporation of more highly-unsaturated fatty acids with ‘bent’ alkene chains (versus ‘straight’ chains of saturated alkanes in saturated fatty acids) to maintain high flexibility of cellular lipid bilayers at low temperatures with concomitant low molecular motion [32]. It has been shown that low temperatures increase *Daphnia*’s demand for dietary PUFAs to allow the maintenance of normal physiology [33, 34] and behaviour [35]. Thus, food quality and temperature constitute intertwined factors that influence the expression of different phenotypes, in order to achieve the best possible performance through plastic acclimatory responses.

In particular, the availability of the highly unsaturated ω3-PUFA eicosapentaenoic acid (EPA, C-20:5 ω3) was repeatedly shown to be crucial for *Daphnia* growth and reproduction, via controlled PUFA supplementation experiments [5, 6, 36]. Aquaculture studies have shown reduced fitness and increased inflammatory responses in organisms from higher trophic levels — such as fish — when ω3 fatty acids are limiting [37, 38], therefore it is of global importance to understand the molecular mechanisms triggered by EPA availability. Alarming prospects in connection with the future availability of EPA on a larger scale were proposed by the results of a meta-analysis, which connected higher water temperatures — due to climate change — with reduced primary production of long chain PUFAs [39].

Despite the growing body of evidence underscoring the importance of dietary PUFAs in general — and of EPA in particular — our understanding of the molecular physiology underlying the PUFA/EPA metabolism, and the gene networks responsive to the availability of this critical dietary compound remain very limited. Heckmann et al. [40] conducted an in-silico analysis of the genome of *Daphnia pulex,* which produced the first insights on potential mechanisms that are affected by ω6 – eicosanoids. Proposed candidate genes are involved in signalling pathways deduced from the ω6-PUFA arachidonic acid (ARA, C-20:4 ω6), affecting prostaglandin and leukotriene signalling. These candidates were confirmed in follow-up gene expression studies [41–43]. However, it is important to emphasise that ω6 PUFAs (like ARA) are generally believed not to be inter-convertible into ω3 PUFAs (such as EPA) in metazoans [30], although this has been questioned [44].

In this study, we hypothesise that dietary availability of EPA will affect specific gene networks connected to lipid metabolism, cellular signalling, and immune-regulating pathways; similar to that which has been demonstrated for eicosanoids derived from ω6-PUFAs [40–42]. We aim to unravel the gene networks specific to dietary EPA availability using a single genotype (clone) of *Daphnia magna* as a model system. Since EPA is crucial for acclimation to low temperatures in *D. magna* [35], such gene networks may become particularly visible at lower temperatures. We thus employed a strictly controlled EPA supplementation experiment at two temperatures, in order to characterise gene expression patterns in *D. magna* using RNA-seq. We discuss these results in connection with the animals’ respective growth performance and fatty acid composition. While an earlier study [6] focused exclusively on single target genes whose expression was dependent on dietary EPA availability, we here look for larger scale transcriptomic adjustments driven by different food types and EPA availability, which should yield insights on PUFA-dependent gene regulation networks. Due to the high level of control of the experimental factors, the results illuminate the genetic basis underlying EPA (and more generally ω3 PUFA)-dependent metabolism in this keystone herbivore; these findings offer important insights for the wider field of herbivore ecology and physiology.

## Results

### Physiological effects at whole animal level

In our experiment, we fed *D. magna* with two different basal diets (GA – green alga, CY – cyanobacteria) that do not contain any long-chain (i.e. > C-18) polyunsaturated fatty acids to monitor physiological and transcriptomic effects of controlled supplementations with the essential C-20 ω3 PUFA EPA.

**Fig. 1:**
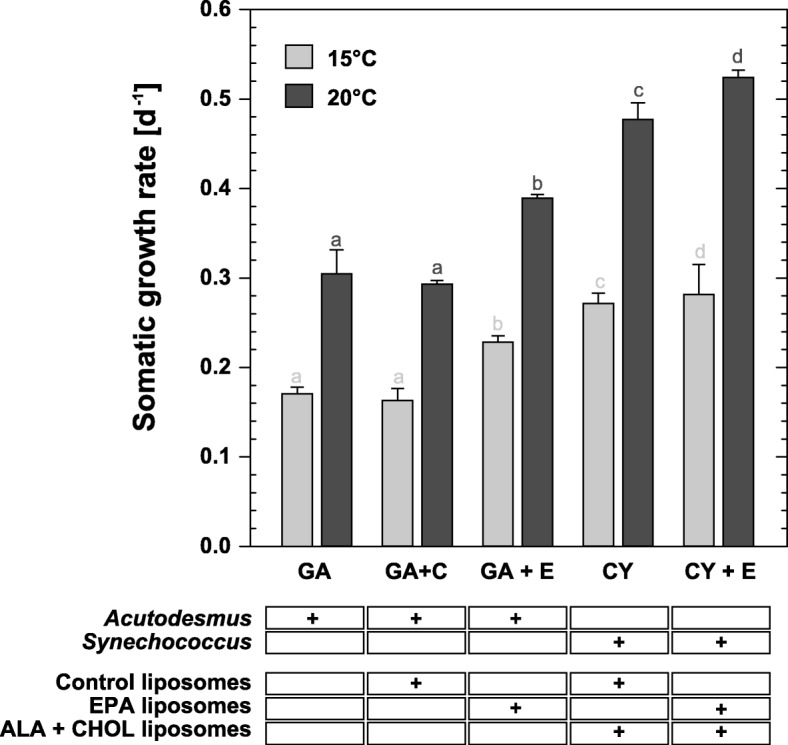
Juvenile somatic growth rates of *D. magna* (means ± SD of *n* = 5) in response to different food sources and EPA-supplementation via liposomes at two experimental temperatures. Different superscript letters indicate significant differences within each temperature regime according to Tukey’s HSD post-hoc tests following a two-way ANOVA. Letters indicate differences (‘a’ is different from ‘b’, ‘c’ is different from ‘d’) within one temperature (by colour); the hash key indicates a difference between the treatments by the factor temperature

Somatic growth rates (SGR) — which are a good fitness proxy in cladocerans [45] — were strongly affected by EPA-availability (2way-ANOVA, *F*_5, 48_ = 411.318, *p* ≤ 0.001) and temperature (2way-ANOVA, *F*_1, 48_ = 2295.402, *p* ≤ 0.001; Fig. 1). Combined effects were also detected (2way-ANOVA, *F*_5, 48_ = 28.779, *p* ≤ 0.001).

In general, growth rates were much lower at 15 °C, reaching only 56.8–61.7% of the performance at 20 °C. EPA had a positive effect on *D. magna* growth when fed with the green alga *Acutodesmus obliquus* at both experimental temperatures (GA + EPA *p* ≤ 0.001). Similarly, EPA improved the SGR when given as a supplement alongside the cyanobacterium *Synechococcus elongatus* (CY + EPA), at 20 °C (*p* ≤ 0.001) and 15 °C (*p* = 0.014).

Somatic growth rates were higher in all CY-treatments than in respective GA-fed cultures. As stated below (see Material and Methods), cyanobacterial diets were further supplemented with cholesterol and alpha linoleic acid in order to support the *Daphnia* to reach maturity (time point of sampling) on this poor diet, which may have enhanced growth rates to the observed levels.

At both temperatures, the supplementation with empty control liposomes (GA + C) had no effect on SGR (20 °C *p* = 0.593; 15 °C *p* ≤ 0.881), indeed similar growth rates were observed when raising *D. magna* on supplement-free food or the respective supplementation of control liposomes to the same basal diet. Although lower growth rates were determined at 15 °C, the animals were up to 26.8% heavier in absolute body mass (data not shown) than the individuals kept at 20 °C.

### EPA incorporation and fatty acid composition

The supplementation of EPA and the natural differences in fatty acid composition in basal diets were considered as main drivers for the observed growth performances — and subsequently for the detected expression profiles — at the respective temperatures.

**Fig. 2:**
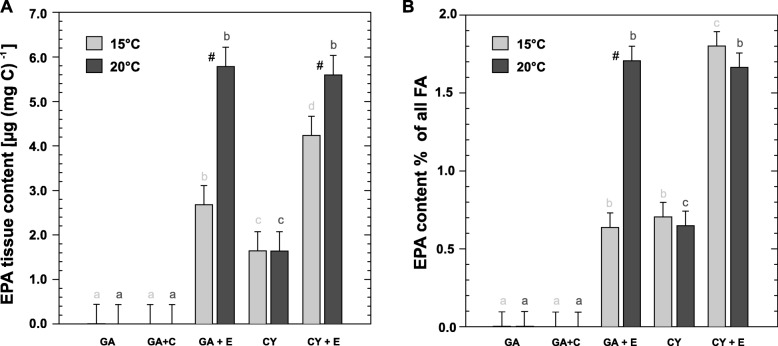
EPA levels in *D. magna*. **a** Mean tissue EPA concentrations (± SEM of *n* = 3 independent replicates consisting of 3 individuals each); **b** mean EPA proportion (± SEM) of all fatty acids. Treatment effects are indicated by different letters, temperature effects by hash keys as determined by two-way-ANOVA followed by Tukey’s HSD *post-hoc* comparisons. Treatment codes indicate basal food item (GA = *Acutodesmus obliquus*, a green alga; CY = *Synechococcus elongatus*, a cyanobacterium) and the supplements (C = control liposomes, E = EPA liposomes)

*D. magna* in EPA treatments accumulated supplemented EPA (Fig. 2a + b). Tissue EPA content of *D. magna* was significantly higher at 20 °C compared to 15 °C.

**Fig. 3:**
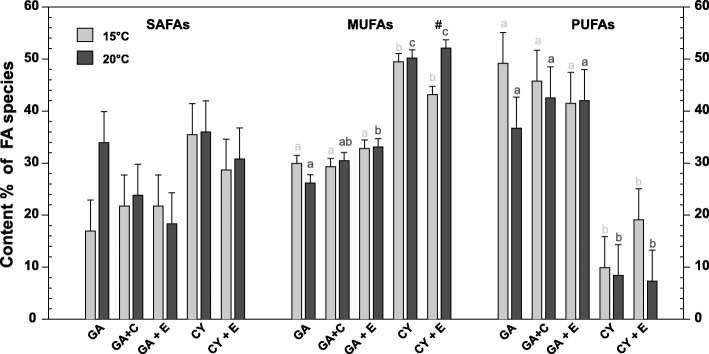
Fatty acid composition of *D. magna* in the experiment. The three groups display the mean (± SEM of *n* = 3) proportion of saturated (SAFAs), monounsaturated (MUFAs) and polyunsaturated fatty acids (PUFAs) in *D. magna* in the respective treatments. Different letters indicate significantly different means within temperatures according to Tukey’s HSD following two-way ANOVA, hash key indicates significant temperature effects. Treatment codes indicate basal food item (GA = *Acutodesmus obliquus*, a green alga; CY = *Synechococcus elongatus*, a cyanobacterium) and the supplements (C = control liposomes, E = EPA liposomes)

The two different basal diets resulted in different tissue fatty acid compositions in *D. magna* (Fig. 3), with respect to the proportions of different fatty acid species (state of saturation). No significant differences were seen for saturated fatty acids (SAFAs), either from the basal diets or from the applied treatment conditions. However, monounsaturated fatty acid (MUFA) proportions differed significantly between diets. At 15 °C, higher MUFA contents were found in CY-fed daphnids (for CY vs GA, GA + C and GA + E *p* < 0.001; for CY + E vs GA, GA + C *p* < 0.001; and CY vs GA + E *p* = 0.002). Similarly, higher contents were detected at 20 °C (for CY as well as for CY + E vs GA, GA + C and GA + E *p* < 0.001). A temperature effect of differing MUFA level was only detectable in the treatment CY + E with (*p* < 0.001).

Polyunsaturated fatty acids (PUFAs) were found to be significantly higher in GA-food sources (all single GAs vs all single CYs *p* < 0.001), with a tendency of higher recruitment at lower temperature, although this result was not significant.

### Differential gene expression overview

**Fig. 4:**
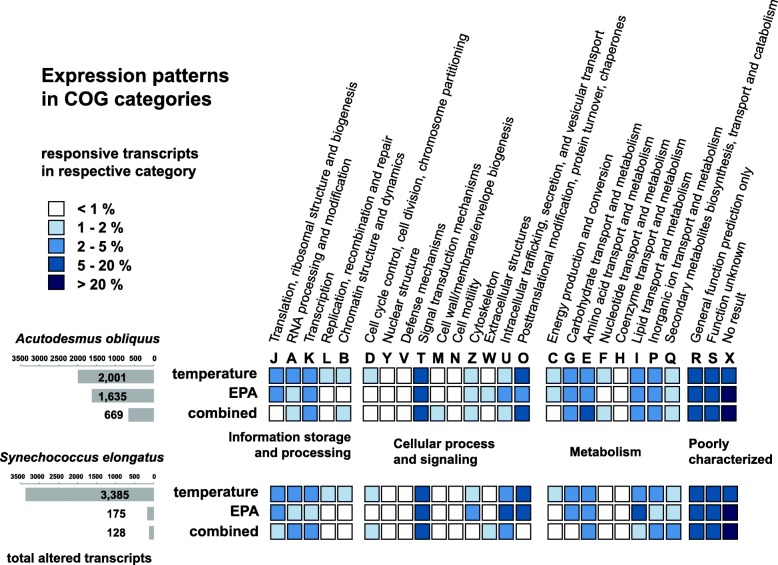
Differential gene expression analysed within basal diets. Display shows a result summary of a two-way ANOVA (at significance level of *p* = 0.01) among expression profiles with the factors ± EPA and ± temperature within basal food types. Grey bars on the left show the amount of significantly different expressed transcripts that were found to be modulated either by temperature, food or combined effects. The total amount of the respective transcripts was then functionally annotated by the ArtNOG categorisation (given in % of the total response). Colour coding indicates the abundance of transcripts in each category

Reads with “poor quality” were excluded (approved by FastQC analyses [46]). The total sequencing output of all samples was 1540.9 million reads, with an average read amount of 51.4 million reads (± 4.1 SD) per sample (see Additional file 1: Table S1). We did not detect differences in the total expression output among treatments or temperatures, thus sequencing depth of the samples was comparable. With a mapping success of 79.89% (± 0.69% SD), we calculated FPKM values for further analyses for each replicate. To broadly compare expression profiles in terms of differential expression driven by food composition and culture temperature, we used the ArtNOG annotation to analyse the transcript diversity among different treatments (Fig. 4) by functional COG (categories of orthologous groups) assignments.

We distinguished differential responses by altered transcripts in *D. magna* fed either GA or CY. In general, the total amount of altered transcripts (driven by EPA, temperature and combined effects) was slightly different, with 3688 and 4305 altered transcripts for GA and CY, respectively. However, temperature-sensitive transcripts were much more pronounced when *D. magna* were raised on cyanobacteria (3385 temperature-specific sequences), whereas on the green algal diet far fewer transcripts (2001 sequences) were altered. The opposite trend was seen for transcripts that displayed EPA-sensitivity: GA treatments yielded 1635 alterations, whereas CY treatments showed only 175 differently expressed transcripts. Similarly, combined effects were more pronounced in GA diet (669 transcripts; CY: 128 transcripts). For both basal diets, altered expression levels were most prominently detected in categories (with known functions) T and O, i.e. ‘Signal transduction mechanisms’ and ‘Posttranslational modifications’, in connection with the factors of temperature and EPA availability. Further changes in cellular processes and signalling categories were seen for ‘Cytoskeleton’ (Z) and ‘Intracellular trafficking, secretion and vesicular transport’ (U). Affected metabolic functions concerned ‘Carbohydrate- ‘(G), ‘Amino acid-’ (E), ‘Lipid-’ (I) and ‘Inorganic ion transport’ (P), as well as ‘Secondary metabolite biosynthesis, transport and catabolism’ (Q). These alterations were paralleled by changes in the ‘Transcription machinery’ (K), as well as alterations in ‘Translational-’ (J) and ‘RNA processing’ (A) transcripts, which were strongly affected by the factor temperature.

The significant gene sets were plotted as volcano plots with a LOG-2-fold change of at least 1 against the adjusted *p*-values to give a general overview on the strongest responses (see Additional file 5 and respective data in Additional file 6).

### Core response profiles of affected transcripts

To provide a more detailed overview of the large set of responsive genes depicted in Fig. 4, we further analysed genes in the respective categories to extract common responses in connection with the factors: temperature, EPA availability, and combined effects of both factors. From the most prominent categories, we cross-matched congruently regulated transcripts in GA and CY treatments to obtain basal diet-independent gene expression patterns (Table 1 and Additional file 2, Additional file 3, Additional file 4).

**Table 1:** Overview of altered transcripts in COG regulated independent from basal diets. Two-way ANOVA results of *D. magna* expression profiles were cross-matched [47] between GA and CY diets to determine common gene expression patterns in functional COG groups. Resulting transcript numbers are given in connection with the respective factors

COG cluster	COG	Category description	Temperature	EPA	Interactions
Information storage and processing	J	Translation, ribosomal structure and biogenesis	31	2	0
	A	RNA processing and modification	54	0	0
	K	Transcription	27	0	0
Cellular processes and signalling	T	Signal transduction mechanisms	50	2	1
	Z	Cytoskeleton	48	1	0
	U	Intracellular trafficking, secretion, and vesicular transport	9	1	0
	O	Posttranslational modification, protein turnover, chaperones	44	1	0
Metabolism	G	Carbohydrate transport and metabolism	17	0	0
	E	Amino acid transport and metabolism	39	1	1
	I	Lipid transport and metabolism	22	0	0
	P	Inorganic ion transport and metabolism	20	1	0
	Q	Secondary metabolites biosynthesis, transport and catabolism	20	0	1
Poorly characterised	R	General function prediction only	100	2	1
	S	Function unknown	97	2	0
	X	No match in artNOG	104	2	1
Total			682	15	5

In total, we found 381 transcripts with a specific functional artNOG assignment that were affected by temperature (details in Additional file 2). The strongest altered gene expression was detected in the cluster of ‘Information storage and processing’, indicating a transcriptomic remodeling driven by temperature. Most of the genes were up-regulated at 15 °C when compared to 20 °C. This may not only be provoked by the necessity of different functions, but also by compensation to maintain efficient reaction norms through increased transcript amounts at lower temperatures. To a lesser extent, this holds also for the COG clusters ‘Cellular processes and signalling’, as well as for genes in ‘Metabolism’ with more complex patterns. Here, functional changes became visible that were not thoroughly connected to compensation strategies. The overall increments in gene expression profiles also varied with the applied basal diet, often with higher expression levels in GA diets than in CY. Interestingly, most temperature-responsive genes of all clusters display a generally higher expression level when EPA was available (see Additional file 2). Specific expression profiles will be detailed and discussed below with respect to the functional patterns.

Far fewer genes were detected for a common response to EPA (15 candidates) or in connection with combined effects (5 candidates; see Table 1 and Additional file 3). The selection of shared altered transcripts between basal diets did include candidates with very different levels of transcript amount.

Many EPA-influenced genes displayed a down-regulation with supplementation, in particular on the GA diets. An exception to this are the transcripts of the carboxylic ester hydrolase and the aromatic-L-amino-acid decarboxylase, which were expressed at the highest levels in animals on GA diets supplemented with EPA. The first may be attributed to lipid metabolism — although jet assigned with artNOG category “R – functional prediction only” — the second is part of amino acid metabolism, and is involved in cell communication and signalling, as this enzyme catalyses the production of dopamine, serotonin, tryptamine, and histamine.

In animals fed CY-EPA diets, the highest expression levels were observed for endo-beta-1.4-mannanase, animal haem peroxidase, and THAP domain-containing protein, which are involved in fructose-mannose metabolism, cyclooxygenase activity, and the regulation of transcription, respectively. Here, a contrasting regulation of transcripts between the different basal diets and EPA supply becomes very explicit.

Genes regulated congruently in both basal diets were Myosin–IB, an uncharacterised protein (KZS03735.1), Angiopoetin-1 receptor-like protein, and Glycerol ether metabolic process (protein); with a down-regulation while EPA is available.

Our statistical analysis, followed by a cross-match of significant genes between diets, yielded six genes that display combined effects of temperature and EPA availability (see Additional file 3). The highest expression level was detected for Cytochrome P450. At the higher temperature, this enzyme was upregulated in the CY + EPA diet, and at the lower temperature in the GA + EPA regime. Transcripts of (putative) Trypsin-7, Endo-beta-1.4-mannanase, as well as Opsin Rh6, were similarly regulated, with higher levels at lower temperature in CY + EPA diets, and were repressed at the higher temperature in GA + EPA diets.

## Discussion

We studied transcriptomic effects of dietary EPA availability in combination with temperature to disentangle responsive gene networks underpinning the beneficial effects of this long chain ω3-PUFA on a physiological level. We further explore these effects by quantifying somatic growth rates as a fitness proxy, together with the animals’ fatty acid composition. This allows us to discriminate gene expression patterns indicative of a complex interplay between resource availability and temperature responses in the aquatic model herbivore *Daphnia magna*.

### Physiological performance and fatty acid composition

As for most animals, the fatty acid composition of *Daphnia* sp. reflects the composition of their diet [48]. In nature, the occurrence of PUFA-rich phytoplankton in lakes at cooler temperatures in spring matches the nutritional demand of zooplankton at the beginning of this season, providing high proportions of PUFAs for growth and reproduction [49], as well as for membrane remodelling [32]. Seasonal shifts in temperature and food availability should therefore be mirrored in altered transcript expression with signatures that are particularly attributable to these factors.

In our analysis, responses in life history traits in connection with EPA availability at different temperatures — demonstrated by impaired growth when EPA was limiting (Fig. 1) — showed that *Daphnia* cultivated at 15 °C displayed a higher demand for EPA than specimens at 20 °C, which is in line with the findings of an earlier study [50]. However, EPA levels of *D. magna* were higher at 20 °C (Fig. 2), contrary to the assumption that more EPA should be required at 15 °C for homeoviscous adaptation. Similar results have been found previously for the same temperature regime [50, 51].

The total amount of EPA as a proportion of total body mass in *D. magna* is higher at the lower temperature. Nevertheless *D. magna* may have been ultimately limited by EPA availability due to the enhanced PUFA demand at lower temperatures. A higher amount of EPA accumulation in somatic tissue at 20 °C than at 15 °C is further supported by a recent study [51].

Overall, when we analysed the daphnids’ fatty acid (FA) composition with respect to saturation state (SAFAs, MUFAs and PUFAs; see Fig. 3) almost no temperature-effects were visible within the different food types (except for MUFAs in the CY + EPA treatment). Consequently, it is likely that the applied thermal difference of 5 °C was not severe enough to alter the animals’ FA contents.

### Gene expression

By assessing gene expression profiles in *D. magna* under strictly controlled experimental conditions, we were able to attribute particular functional changes specific to temperature and EPA availability. In general, temperature elicits large responses connected to RNA and DNA related processes (“Information storage and processing”, see Fig. 4), which are represented by a complex network of genes involved with replication as well as with transcription and translation. This key abiotic factor also provoked the alteration of transcripts that affect signal transduction mechanisms, posttranslational modification, as well as carbohydrate-, amino acid- and lipid transport mechanisms, and inorganic ionic transport processes.

Although the effect size of EPA altered transcripts was lower than the temperature-induced effects, this dietary constituent is nevertheless a major driver of improved growth at the physiological level.

The transcriptomic responses so far analysed in connection with long chain polyunsaturated fatty acids rely on studies of enzymes that are involved in eicosanoid synthesis of the “arachidonic pathway” [52]. These enzymes are known to convert eicosanoids into important signalling molecules, such as prostaglandins or leukotrienes in invertebrates, but also in mammals [40, 53]. In our study, EPA availability provoked various functional changes in translation and transcription, but also in signal transduction mechanisms, changes in intracellular trafficking, as well as altered transcript levels for carbohydrate-, amino acid-, and lipid metabolism that are detailed below.

### Information storage and processing

In this cluster, the strongest thermal effects are seen for the categories ‘RNA processing and modification’ (category A); ‘translation, ribosomal structure and biogenesis’ (J), and ‘transcription’ (K). Adjustments in the transcriptome become visible here, as these functions are modulated as a first response to the altered conditions. A high proportion of maintenance costs is attributed to regulation of this gene, which compensates effects of bio-physical reaction norms [54–56]. Generally, higher expression values were observed at the colder temperature, and were more enhanced than in other functional classes, such as ‘cellular processes and signalling’ or ‘metabolism’ (see Additional file 2). This effect is known as the compensatory effect, and was previously shown to vary between clones of *D. pulex* due to local adaptation [57]. Many candidates attributed in this cluster through artNOG annotation showed high transcript levels at both lower temperature and dietary EPA availability. This indicates adjustments of the transcriptome in response to changes in both factors.

### Signalling

Numerous G-protein signalling transcripts, as well as serine/threonine kinases and opsins, were found to be thermally sensitive and were elevated with dietary EPA availability. Such candidates are potential mediators of anti-inflammatory processes, and are connected to healing and growth of cells in mammals [58]. Further, RAs and Ran- transcripts (RAs-related nuclear protein) — that are factors involved in G-protein signalling affecting gene expression cascades involved in cell growth, differentiation and survival [59] — were upregulated. It is likely that RAs and Ran transcripts mediate sensing and signalling cascades for growth in *Daphnia* and other invertebrates. Similarly, the production of resolvins and protectins, molecules derived from EPA as well as from the longer docosahexaenoic acid (DHA, 22:6 ω3), are involved in cytokine and leukotriene signalling via G-proteins [60]. Transcripts of signalling cascades involving stimulators like dopamine or serotonin (products of the aromatic-L-amino-acid decarboxylase) were found to be upregulated in the EPA treatments. It remains to be investigated whether such products do function as neurotransmitters, or if they serve other endocrine functions in invertebrates. The first indication for the utilization of dopamine in *Daphnia* sp. was found in connection with predatory stress [61].

We also identified transcripts of cytochrome P450 in connection with the combined effects of temperature and EPA availability (Additional file 3). This is an important indicator for the biotransformation of EPA. Potential mechanisms are the conversions of EPA into five regioisomeric epoxyeicosatetraenoic acids (EETeTrs) and ω/(ω-1)- hydroxyeicosapentaenoic acids (19- and 20-HEPE) [62], which mediate (at least in mammals) a delicate balance between pro- and anti-inflammatory responses [63].

Numerous gene families of cytochrome P450, as well as pseudogenes, have been identified across the animal kingdom [64]. For *Daphnia*, 75 functional CYP genes and 3 pseudogenes belonging to 4 clans, 13 families, and 19 subfamilies are so far identified [65]. However, the particular functional implications for many of these genes are still to be determined.

### Cellular structure and metabolism

The higher growth rates were paralleled by higher expression of genes for cytoskeletal structures, accompanied by induced growth factor receptors and fibronectin, which were expressed at higher levels at 15 °C when EPA was available.

The different profiles of carbohydrate metabolic transcripts (G, but also in E) maltase, amylase and alpha-glucan branching enzymes indicate a different quality of the basal food sources, as well as different energetic demands at both temperatures. Whilst sugars seem to be strongly metabolized at 20 °C, glycogen anabolism becomes more effective at 15 °C. This may be due to the fact that a faster metabolism is connected to a higher temperature, and is thus accompanied by a higher demand for sugars; this is mirrored by higher growth rates at the physiological level. Carbohydrate metabolism was also differentially regulated when *Daphnia sp.* were challenged with diets of different qualities in terms of nutrient stoichiometry [66], which may indicate that this is a very general response to food quality alterations. Genes in carbohydrate metabolism involved in inflammatory processes (SAPA), or connected to chitin and moulting (chitotriosidase), were also regulated in a temperature-dependent manner; these do however reflect a higher variation that may mirror the variability of individuals in sample pools.

Despite low transcriptional levels in the GA-diet regime, peptidases like trypsin or chymotrypsin, aminotransferases and metallopepdiases were up-regulated in CY diets, especially at the lower temperature (E). This was also mirrored in the expression of Eip55E (Ecdysteroid-inducible polypeptide 55 subunit E), which is involved in sulphur amino acid metabolic processes like cysteine and glutathione biosynthesis.

The different expression profiles in carbohydrate and amino acid metabolism indicate a recruitment of different enzymes to extract energetic compounds like sugars or amino acids from the different basal diets [67]. Different digestive efforts for CY-diets are indicated through high expression levels of metallopepdidases, trypsins, and aminotransferases, as well as by chaperones like T-complex proteins. Therefore, different basal diets provoke different phenotypes to handle and digest the different food items.

In the ‘lipid metabolism’ category (I), high levels of acyl-CoA dehydrogenases were expressed at 20 °C, especially when EPA was available. This indicates the transcription of RNAs related to the degradation of fatty acids. Transcript levels for transporters and intracellular transport structures associated with the transport mechanisms of long chain fatty acids were up-regulated when EPA was absent. This may be a mechanism to cover the higher demand for long chain PUFAs under EPA limitation. The higher expression levels of fatty acid transporters was accompanied by the expression of a transporter in the category ‘inorganic ion transport and metabolism’ (P), as well as by ABC transport proteins (ATP-Binding Cassette sub-family C/ member 4) and cytochrome P450 305a1, which are involved prostaglandin-mediated signalling (Q, secondary metabolites).

High vitellogenin levels were pronounced in GA + EPA diets, accompanied by the expression of glycerol-3-phosphate acyltransferase and acyl-CoA-binding domain-containing protein 7, with slightly higher (perhaps compensatory) levels at 15 °C, this may be involved in the biogenesis of vitellogenin, as previously observed [43]. Further, a secretory phospholipase A2 was induced, indicating increased effort to digest liposomal-supplemented diets.

High levels of dynein, myosin, and tubulin (Z) indicate a remodelling of the cytoskeleton at lower temperatures. As the solubility and viscosity of the cytosol seems to be affected, a structural remodelling is indicated by the latter transcripts that are further supported by EPA availability. In this context, higher levels of fibronectin and endothelial growth factor receptor indicate a mediation of processes involved in cell division and growth [68].

Expressed candidate genes connected to ‘inorganic ion transport and metabolism’ (P) were up-regulated in animals feeding on cyanobacteria at 20 °C. Further gene upregulation occurred at 15 °C, which indicates dynamic adjustments of the osmotic balance at the lower temperature. Ca^2+^- and serotonin transporters were particularly more strongly expressed at the lower temperature in the GA- diet supplemented with EPA. This matches the observed pattern for G-protein transcripts and conjoined candidates in category T, which may contribute to the same messaging pathway [69]. Similarly, cytochrome P450 305a1 transcript displayed the same pattern in the category Q ‘secondary metabolites…’, which may also indicate a conjoined function in a signalling pathway. Interestingly, other Cytochrome P450-like proteins seem to be highly temperature sensitive, and were expressed with low or high levels in GA + EPA diets at 20 °C and 15 °C, respectively.

Cytochrome P450 transcripts and subsequent proteins seem to play an important role in the metabolism and potential transformation of EPA into signalling cascades. Potential pathways for a transformation of the long chain polyunsaturated fatty acid EPA into other endocrine signalling molecules were proposed by [40, 53], these are: the cyclooxygenase (COx) pathway; 2) the lipoxygenase (LOX) pathway; or 3) the cytochrome P450 pathway. Recent expression studies, however, have shown that COx expression is not affected by EPA-availability [43, 52], and so far, no LOX genes have been found in *Daphnia* species [43]. Overall, our study delivers a profound insight into EPA-connected metabolism, and indicates that a transformation into endocrine signalling may rely on Cytochrome P450-based conversions; these outcomes should be explored in detail by further studies.

## Conclusions

This study illuminates the plastic transcriptional responses in *Daphnia magna* to different food types and temperature regimes. The expression patterns in different phenotypes were highly dependent on the dietary availability of the ω3-polyunsaturated fatty acid EPA. Our results suggest a distinct cascade effect, utilizing different forms of cytochrome P450 to mediate sensing the essential EPA compound by transformation and subsequent G-protein signalling. Affected target genes then stimulate further transcription, upregulate transport mechanisms of intermediates, and stimulate cellular growth and reproduction. Altogether, this promotes a positive overall physiological performance. This is the first time that the orchestrating gene responses of *Daphnia* to these stimuli are explicitly demonstrated. Our study thus reveals some of the molecular mechanisms underlying the positive effects of a particular dietary omega-3 fatty acid, and constitutes an important resource of transcriptional patterns, even in genes with little or no annotation. Further, we propose that many new candidates for future investigations on the characterisation of EPA-related pathways are to be found in the currently uncharacterised genes (see expression profiles: Additional file 4), which can be now be annotated to be EPA-responsive.

Since cladoceran zooplankton forms the link in the trophic transfer of matter and energy between primary producers to higher levels in the food chain, this is an important step for our understanding of resource-driven limitation at the aquatic plant-herbivore interface.

## Methods

### Food cultures

The green alga (GA) *Acutodesmus obliquus* and the cyanobacterium (CY) *Synechococcus elongatus* served as food sources. The diets were chosen to monitor effects of the fatty acid EPA via supplementation, as they do naturally not contain long chain (> 18 C) ω3 PUFAs [50, 70, 71].

Respective culture conditions of all used food sources are listed in Table 2.

**Table 2:** Algal and cyanobacterial culture conditions and respective growth media

Organism	Strain	Medium	Culture conditions	Temperature and light conditions
Green alga *Acutodesmus obliquus*	SAG 276-3a	Z/4 [72]	5 L semi-continuous batch culture with a 20% Vol. replacement with fresh sterile medium every other day	20 °C and a constant light intensity
Cyanobacterium *Synechococcus elongatus*	SAG 89.79	Cyano [73]	chemostat at a dilution rate of 20% d^−1^	of ~ 60 μmol photons m^−2^ s^−1^

### Animals and experimental design

*Daphnia magna* originating from the pond Driehoek in Heusden (clone P132.85; The Netherlands; N51°44′01″, E5°08′17″) were used for the experiments. Aerated tap water was aged for at least 2 days and was used as culture medium after sterile-filtration (45 μm). The animals were kept under dim light conditions and were pre-cultured at either 15 °C, or 20 °C for at least 2 months. A maximum density of 15 individuals L^− 1^ was not exceeded. Feeding was based on green algal diet (*A. obliquus*) with a standardized amount of 2 mg carbon L^− 1^ every second day during pre-culture.

Juveniles for experiments originated from the third clutch (parthenogenetic offspring) and were collected within 8 h after release from the mothers’ brood pouch. After pooling, neonates were randomly distributed into vessels containing 600 ml tap water with experimental diet and were either incubated at 20 °C or 15 °C. The exposure medium with food and supplements was exchanged on a daily basis (20 °C) or every other day (15 °C).

Food treatments comprised a green algal (GA), or cyanobacterial (CY) basal diets, as well as supplemented treatments with EPA liposomes (GA + EPA, CY + EPA). To control that liposomes themselves had no effect we included a control (GA + C) supplementing the empty vector. All conditions were replicated with *n* = 5. Each replicate comprised 15 individuals.

Liposomes were prepared as according to Martin-Creuzburg et al., 2008 [74]. Individuals were sampled when they reached maturity, indicated by the deposition of the first clutch of eggs into their brood pouch. As *D. magna* requires a minimum of PUFASs and sterols to produce offspring [8], cyanobacterial diets were further supplemented with at alpha linoleic acid (ALA, C-18:3 ω3) and cholesterol to ensure the reach of the sampling end-points. The total supplementation loading for all treatments was standardized to a concentration of 320 μl liposomes L^− 1^.

Sampling was carried out by picking single individuals, rinsing them twice with deionized water, blotting them dry with lint-free tissue and shock-freezing them by use of liquid nitrogen. Samples were stored at − 80 °C until extraction of RNA or fatty acids. Two individuals from each vessel were used for somatic growth rate analysis.

### Daphnia fitness parameters

Somatic growth rates were monitored using the mean dry weight of two adult individuals from each replicate vessel when reaching maturity. As a reference we used the mean dry weight of 40 neonates at the beginning of the respective experimental temperature. All collected animals were rinsed with deionized water and then dried for 24 h at 60 °C. Drying and mass determination was done in (pre-weighed) aluminium boats. Somatic growth rates (SGR) were determined in accordance to Lampert et al. [45] to monitor whole animal fitness by the formula:

$$ SGR=\left[\right(\ln \left({m}_t\right)-\ln \left({m}_0\right)\Big]/t. $$

As *Daphnia* grows with an exponential rate, the natural logarithms were applied to final weights m_t_ from each replicate and to the starting weight m_0_ to calculate the net weight gain by their difference. Growth rates were than put into relation to the time period t, the number of days until reaching maturity, which was indicated by formation of the first clutch of offspring in their brood chamber [75].

### Fatty acid composition

The analysis of fatty acid composition was done by means of fatty acid methyl esters (FAME), which were subsequently quantified by gas chromatography (GC). For each treatment 3 mature *D. magna* from each replicate (previously stored at − 80) were extracted with 5 ml extraction reagent (ExR, dichloromethane/methanol (2:1, v:v)) over night at 4 °C. Internal standards with uneven length of the fatty acid chain, which do not naturally occur in *Daphnia* or Alga, were added to the extraction as a mass reference: 10 μg heptadecanoic acid methyl ester (C-17:0 ME) and 5 μg tricosanoic acid methyl ester (C-23:0 ME). The extract was mixed on a vortexer and then sonicated for 1 min. The extract was collected using a glass Pasteur pipet and another 3 ml ExR were added to the tube containing the daphnids. After a second round of vortexing and sonication extracts were pooled and centrifuged at 5.000 rpm for 5 min at room temperature to remove cellular debris. The solvent was evaporated under a constant stream of nitrogen until the sample was dried. By adding 5 ml of 3 N methanolic HCl and incubation at 70 °C for 20 min the sample was transesterified to built FAMEs. Extraction of FAMES was carried out by adding ~ 2 ml isohexane, vigorously vortexing for a minute and collection of the upper liquid phase. The remaining phase was again extracted with isohexane to minimize loss FAMES that were not transferred in the first round. After solvent evaporation, FAMEs were collected from the glass vial by pipetting 3 × 100 μl, rinsing the walls of the vial. The extract was collected in a HPLC-vial. Again, the solvent was evaporated and FAMEs were finally dissolved in 50 μl isohexane to concentrate and standardize samples for the GC measurement. For all measurements 1 μl FAME-extract was analyzed on a 6890-N GC System (Agilent Technologies, Waldbronn, Germany) equipped with a DB-225 capillary column (30 m, 0.25 mm i.d., 0.25 μm film thickness, J&W Scientific, Folsom, CA, USA). The instrument was run with the following settings: injector and FID temperatures were 200 °C; the initial oven temperature was 60 °C for 60 s, followed by a thermal ramp to 180 °C with 2 °C/s, then a ramp to 200 °C with 0.83 °C/min followed by 630 s at 200 °C, followed by ramp to 220 °C with 2 °C/s followed by 630 s at 220 °C; helium with a flow rate of 0.025 ml/s was used as the carrier gas. The fatty acids were then quantified by referring to the mass and signals of internal standards and to standards for each FAME from mixtures of known composition [5, 76]. Each calibration for single FA had a correlation coefficient > 0.98. Single fatty acid contents were related to the carbon content of the body tissue using the previously determined carbon to dry mass conversion factor for body tissue, 0.41 μg carbon (μg dry mass)^− 1^ [77].

### Nucleic acid preparation and sequencing

Based on their different fitness at the physiological level, which was monitored by means of somatic growth rates, we selected sample pools for RNA extraction. From both experimental temperatures we prepared RNA from 4 treatment groups for sequencing with 3 replicates each. We did not include the GA (only food) treatment, as there were no differences to GA + C on physiological level (SGR) or in PUFA composition. We pooled the material of 5 specimens for each replicate to ensure appropriate amounts for RNA extraction and comparability. We used the NucleoSpin RNA extraction kit (Machery und Nagel, Düren/Germany) according to the manufacturer’s instructions for total RNA extraction. To ensure, that traces of genomic DNA do not give false signals, we additionally used the Turbo DNA-free Kit (Invitrogen, Karlsruhe/Germany) as an additional clean-up. The RNA-extract was analyzed on a Bioanalyzer (Agilent Technologies, Santa Clara/USA) to determine quality and quantity of the RNA. Samples with OD_280_/OD_260_ ≥ 2.0, OD_280_/OD_230_ ≥ 2.0 and RIN ≥ 8.0 were used for sequencing. The following cDNA library construction, fragmentation as well as sequencing was conducted at the University of Cologne’s ‘Cologne Centre for Genomics’ (CCG). cDNA libraries were built for each sample and subsequently sequenced on an Illumina HiSeq 4000 platform in the paired-end 75 bp –mode. All 30 samples were run in pools (5 treatment groups with 3 replicates at two temperatures) distributed to two lanes. The first raw data cleaning (clipping and trimming) was done by the CCG as well using Trimmomatic [78].

### Data analysis and processing

We sequenced mRNA of pools of 5 *D. magna* individuals from 4 different food treatments (GA + C, GA + EPA, CY, CY + EPA) originating from either 15 °C, or 20 °C with 3 replicates each. We used FastQC [46] to monitor sequence quality at a critical threshold score > 30, which was passed for samples. Reads originating from single treatment replicates were mapped to *D. magna* genome (NCBI BioProject accession No. PRJNA298946, comprised of ~ 130 million bases) to gather single specific expression profiles. The reference database (v2.4) contains 26.646 translations (open access since April 2016). We used RSEM v.1.2.31 [79] connected to bowtie2 [80] for mapping and to generate FPKM values for transcript analysis.

To gather a more functional overview, we assigned the translations of genomic sequence data with orthologous groups using the eggNOG v. 4.0 database [81]. In particular, we assigned sequences with artNOGs (arthropod Non supervised Orthologous Groups, composed of 21 species in this group), which are grouped in COG (Categories of Orthologous Groups). For assignments we used BLASTp at an e-value cut-off ≤10^− 3^ and a HSP cut-off length ≥ 33 bases to provide additional information for categorization.

For differential gene expression analysis we used MeV software v. 4.8.1 [82] connected to edgeR [83]. Two-Way ANOVA was used on the basis of normalized FPKM values to identify differently expressed genes driven by EPA-availability and temperature at respective basal food sources.

In addition, t-tests were conducted between treatments within temperatures, or vice versa characterise gene expression profiles at a critical threshold of *p* ≤ 0.01. Responsive transcripts were analysed via cross-match (Hulsen, de Vlieg, & Alkema, 2008) to gather consensus lists.

### Data validation

To corroborate the RNA-seq data, we examinated the gene expression profiles of five selected candidate and housekeeping genes from our RNA-seq experiment in comparison to qPCR data from literature.

Selected transcripts were assessed in a former qPCR-study by Schlotz et al. [42], who monitored the transcriptomic response of *Daphnia magna* fed with different natural food types. The experiments were carried out at 20 °C and daphnids were fed with either a green alga (*Scenedesmus obliquus*, now *Acutudesmus obliquus*), or a cryptomonad (*Cryptomonas* spec.) until the generation of the third clutch of offspring. Cryptomonads naturally contain numerous omega-3-fatty acids, including EPA.

Schlotz et al. observed increased levels of vitellogenin [42] when the EPA-rich *Cryptomonas* spec. Was fed to *D. magna*. We made similar observations in a recent feed supplementation study [43], where *D. magna* fed with a green alga (*Acutodesmus obliquus*) showed lower levels of vitelloginin transcripts than EPA-supplemented daphnids. The same pattern was detectable in our RNA-seq data. Vitellogenin transcripts (product accessions KZS21011.1, KZS21012.1 and KZS06496.1) were significantly increased (2-way ANOVA) when dietary EPA was available (in the set of “significant to food GA”).

The transcript for juvenile hormone was another target in the study of Schlotz et al. [42]. They observed increased levels of juvenile hormone transcripts when *D. magna* were fed the EPA-rich food source *Cryptomonas* sp. The same pattern was detectable in our RNA-seq data (product accession KZS09289.1). Juvenile hormone was significantly increased (2-way ANOVA) when dietary EPA was available to *D. magna*.

Fatty acid binding protein 3 was another candidate gene derived from the study of Schlotz et al. [42]. They found a down-regulation when *D. magna* were fed the EPA-rich food source *Cryptomonas* sp. We detected the same pattern in our RNA-seq data when dietary EPA was available to *D. magna* (product accession KZS18357.1).

We further compared the expression of two housekeeping genes in our RNA-seq data (from the set “food GA non-significant”) with qPCR expression data from earlier, published studies: Schwarzenberger et al. [84, 85] and Windisch and Fink [6] used glycerinaldehyde-3-phosphate dehydrogenase (GAPDH) for normalisation of qPCR data. Respective transcript levels in our RNA-seq data (product accessions KZS15286.1, KZS06031.1, KZS02020.1, KZS01487.1, KZS01268.1, KZS98609.1, KZR98522.1) also displayed only minor variation among treatments, thus corroborating the house-keeping character of the target gene.

Similarly, Succinate Dehydrogenase (Suc-DH), used in the studies mentioned above [84, 85], did not appear to be under differential regulation under the different treatments of our study (product accessions KZS06688.1, KZS04241.1, KZR98715.1, KZS19960.1, KZS19824.1). Taken together, our RNA-seq data match published expression pattern from literature determined via qPCR. This hence corroborates that our expression profiles determined via RNA-seq are representative and comparable to other methodologies and studies.

## Supplementary information

**Additional file 1: Table S1.** Summary of sequencing output and mapping success.

**Additional file 2.** Temperature significant gene expression profiles.

**Additional file 3.** EPA significant gene expression profiles and combined effects.

**Additional file 4.** Expression profiles of “poorly characterized” genes responsive to temperature, EPA and combined effects.

**Additional file 5: **Volcano plots of transcripts with LOG2-fold changes > 1 (identified by two-way ANOVA, based on means of triplicates in each group). Respective values were plotted against the adjusted *p*-values. A respective COG-annotation of single transcripts was included by colour of the data points. Single data points were also labelled with product accession numbers for identification. The underlying data sets were exported as Additional file 6.

**Additional file 6:** Datasets of responsive gene sets with LOG2-fold change values, adjusted p-values and the respective COG category.

## Data Availability

RNA-seq data are available at the NCBI sequence read archive under accession number SRP109969 with sample accessions SAMN07259933 - SAMN07259947 (20 °C) and SRS2947545 - SRS2947559 (15 °C). A detailed description and overview of the respective sampling material is available under the BioProject accession number PRJNA391248. The results of the RNA-seq experiment are also accessible under the GEO entry GSE130674.

## Abbreviations

+ C: Supplementation with control liposomes
+ E or EPA: Supplementation with EPA liposomes
ALA: Alpha linoleic acid, C-18:3 ω3
ANOVA: Analysis of variance
ARA: Arachidonic acid, C-20:4 ω6
artNOG: Arthropod non-supervised orthologous groups
BLAST: Basic Local Alignment Search Tool
bp: Base pair
C-17:0 ME: Standard for FAME analytics, heptadecanoic acid methyl ester
C-18: Fatty acids with total length of 18 carbon molecules
C-20: Fatty acids with total length of 20 carbon molecules
C-23:0 ME: Standard for FAME analytics, tricosanoic acid methyl ester
CCG: Cologne Centre for Genomics
cDNA: Complementary DNA
COG: Categories of orthologous groups
COx: Cyclooxygenase
CY: Cyanobacterium (here in particular of the species *Synechococcus elongatus*)
Cyano: Cyanophycea medium, used for the culture of *Synechococcus elongatus*)
CYP: Cytochrome p-450
DHA: Docosahexaenoic acid, C-22:6 ω3
DNA: Deoxyribonucleic acid
EETeTrs: Epoxyeicosatetraenoic acids
eggNOG: Database for orthologous groups hosed by the EMBL
EPA: Eicosapentaenoic acid, C-20:5 ω3
ExR: Extraction reagent for lipids extraction
FA: Fatty acid
FAME: Fatty acid methyl esters
FastQC: Quality assessment software for sequencing data
FPKM: Fragments per kilobase of exon model per million reads mapped
GA: Green alga (here in particular of the species *Acutodesmus obliquus*)
GC-FID: Gas chromatography-flame ionization detector
HEPE: Hydroxyeicosapentaenoic acids
HSP: High scoring pairs
LOX: Lipoxygenase
MeV: Multi Experiment viewer
MUFA: Monounsaturated fatty acid
NCBI: National Centre for Biotechnology Information
PUFA: Polyunsaturated fatty acid
Ran: Ras-related Nuclear protein
Ras: Protein family involved in G-Protein signalling
RNA: Ribonucleic acid
RNA-seq: Ribonucleic acid sequencing
rpm: Rotations per minute
SAFA: Saturated fatty acid
SAG: International acronym for Culture Collection of Algae at Göttingen University, Germany
SAPA: Substrate-associated protein A
SD: Standard deviation
SEM: Standard error of means
SGR: Somatic growth rate
THAP: Thanatos Associated Protein, indicates death-domain in protein
v:v: Percent of volumes
Z/4: Name of the medium used for *Acutodesmus obliquus* culture
ω3: Fatty acid species with unsaturation (carbon double bond) at the n-3 position
ω6: Fatty acid species with unsaturation (carbon double bond) at the n-6 position
