# Supplementary material for: Transcriptome sequencing of a keystone aquatic herbivore yields insights on the temperature-dependent metabolism of essential lipids
Source: BMC Genomics. 2019 Nov 21;20:894. doi: 10.1186/s12864-019-6268-y (PMC6873670; doi:10.1186/s12864-019-6268-y)
Supplement: Supplementary file 1 — Additional file 1: Table S1. Summary of sequencing output and mapping success. [file 12864_2019_6268_MOESM1_ESM.docx]

Supplementary Tables

**Table 1:** **Summary of sequencing output and mapping success.**

| **Temperature** | **Treatment** | **Number of reads** | **Sequences flagged as poor quality** | **Seq. length** | **% GC content** | **Mapping success** |
| --- | --- | --- | --- | --- | --- | --- |
|  | CY-E #1 | 51493224 | 0 | 35-76 | 46 | 78.79 |
|  | CY-E #2 | 45486580 | 0 | 35-76 | 46 | 79.42 |
|  | CY-E #3 | 48302302 | 0 | 35-76 | 46 | 79.69 |
|  | CY+E #1 | 50584252 | 0 | 35-76 | 46 | 79.18 |
|  | CY+E #2 | 56803308 | 0 | 35-76 | 46 | 78.47 |
|  | CY+E #3 | 50936050 | 0 | 35-76 | 46 | 79.47 |
| 15°C | GA-E #1 | 52530620 | 0 | 35-76 | 46 | 80.12 |
|  | GA-E #2 | 46454188 | 0 | 35-76 | 46 | 80.29 |
|  | GA-E #3 | 54939898 | 0 | 35-76 | 46 | 80.23 |
|  | GA+E #1 | 50156498 | 0 | 35-76 | 46 | 79.99 |
|  | GA+E #2 | 48010156 | 0 | 35-76 | 46 | 79.76 |
|  | GA+E #3 | 47226976 | 0 | 35-76 | 46 | 80.2 |
|  | CY-E #1 | 57429222 | 0 | 35-76 | 46 | 79.34 |
|  | CY-E #2 | 54164982 | 0 | 35-76 | 46 | 80.82 |
|  | CY-E #3 | 50853402 | 0 | 35-76 | 46 | 79.75 |
|  | CY+E #1 | 49917634 | 0 | 35-76 | 46 | 79.89 |
|  | CY+E #2 | 55526990 | 0 | 35-76 | 46 | 79.91 |
|  | CY+E #3 | 58833380 | 0 | 35-76 | 46 | 80.95 |
| 20°C | GA-E #1 | 53185492 | 0 | 35-76 | 46 | 80.14 |
|  | GA-E #2 | 51957976 | 0 | 35-76 | 46 | 80.04 |
|  | GA-E #3 | 50926358 | 0 | 35-76 | 46 | 80.68 |
|  | GA+E #1 | 57620528 | 0 | 35-76 | 46 | 80.55 |
|  | GA+E #3 | 55729748 | 0 | 35-76 | 46 | 80.45 |
|  | GA+E #3 | 45555500 | 0 | 35-76 | 46 | 80.35 |
