# Supplementary material for: Transcriptome sequencing of a keystone aquatic herbivore yields insights on the temperature-dependent metabolism of essential lipids
Source: BMC Genomics. 2019 Nov 21;20:894. doi: 10.1186/s12864-019-6268-y (PMC6873670; doi:10.1186/s12864-019-6268-y)
Supplement: Supplementary file 2 — Additional file 2. Temperature significant gene expression profiles. [file 12864_2019_6268_MOESM2_ESM.pdf]

Information storage and processing

Category A - RNA processing and modification

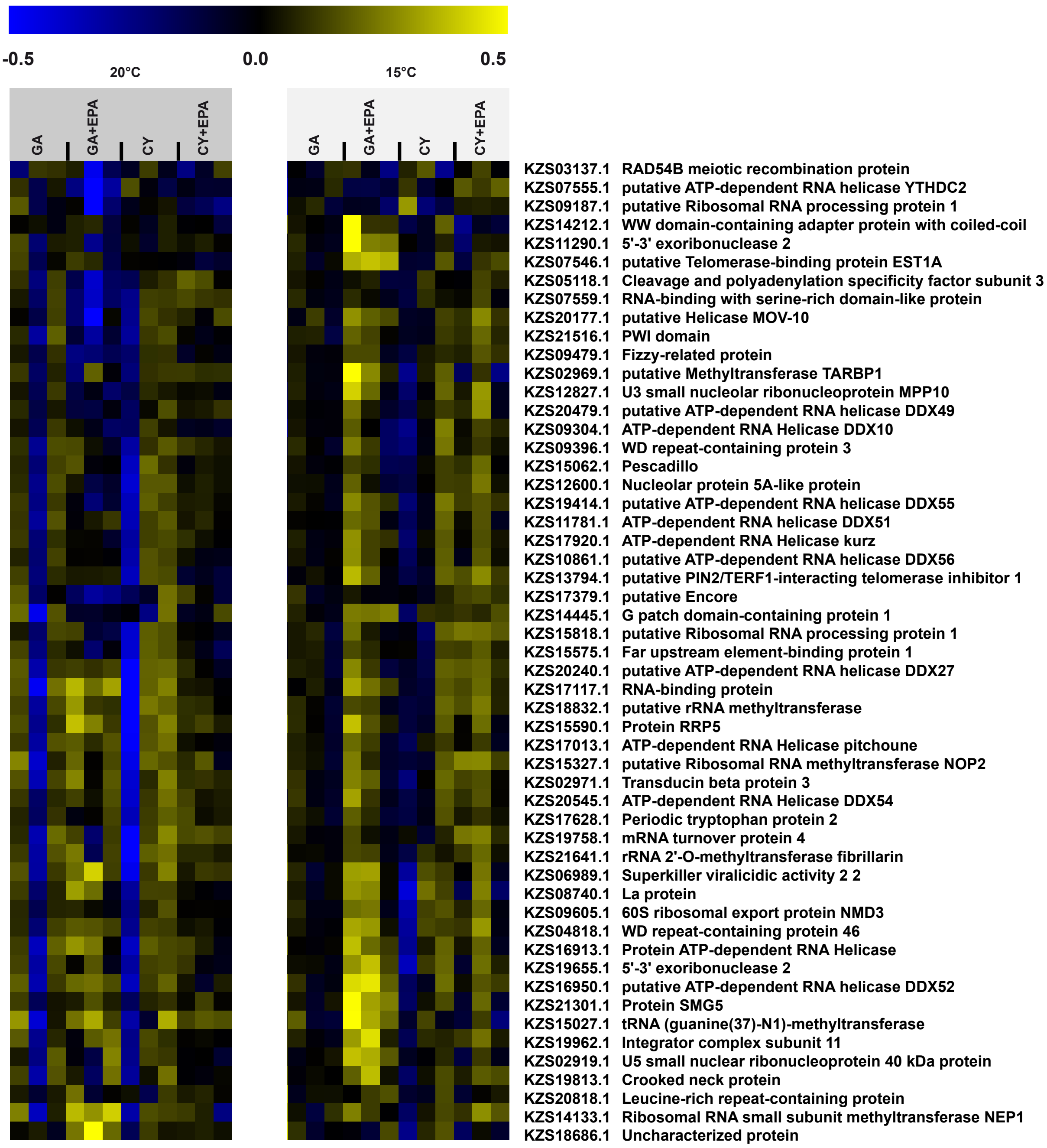

Information storage and processing

Category J - Translation, ribosomal structure and biogenesis

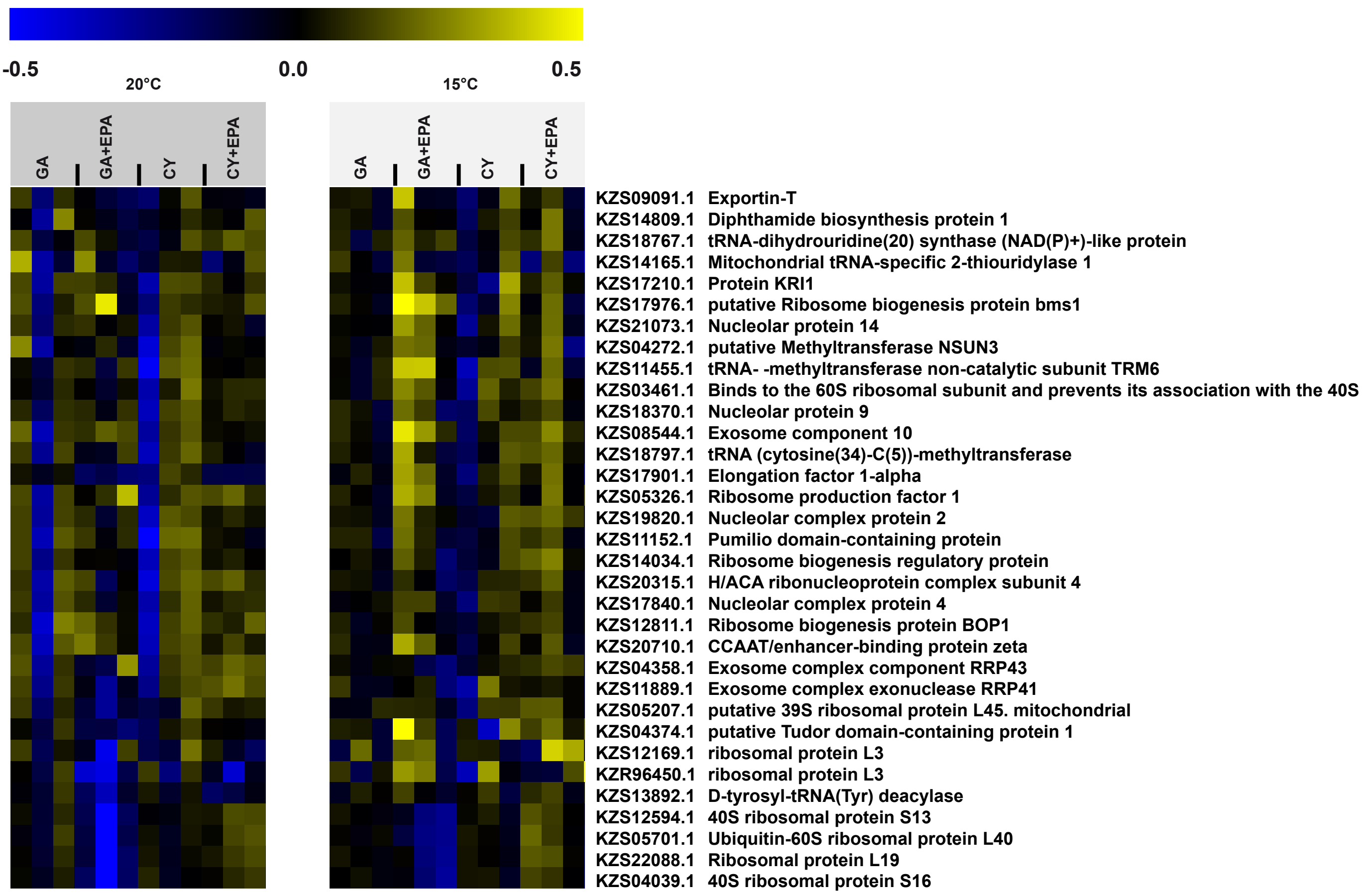

Information storage and processing

Category K - Transcription

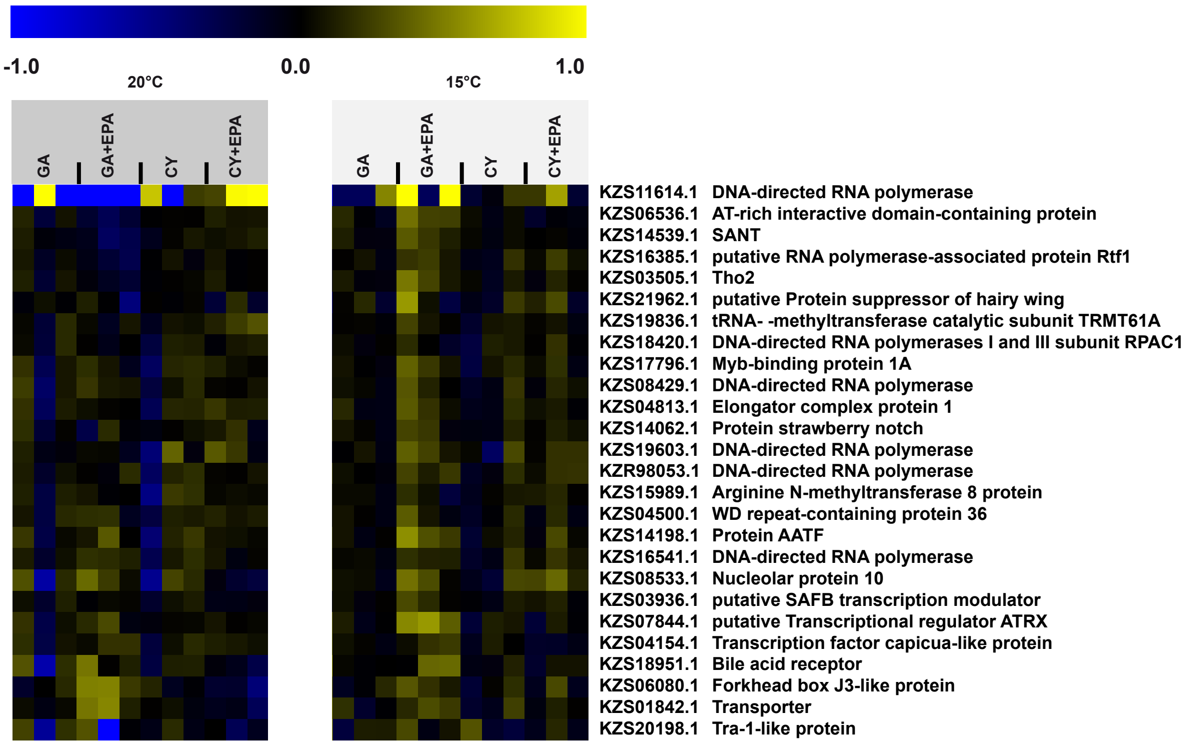

Cellular processes and signalling

Category O - Posttranslational modification, protein turnover, chaperones

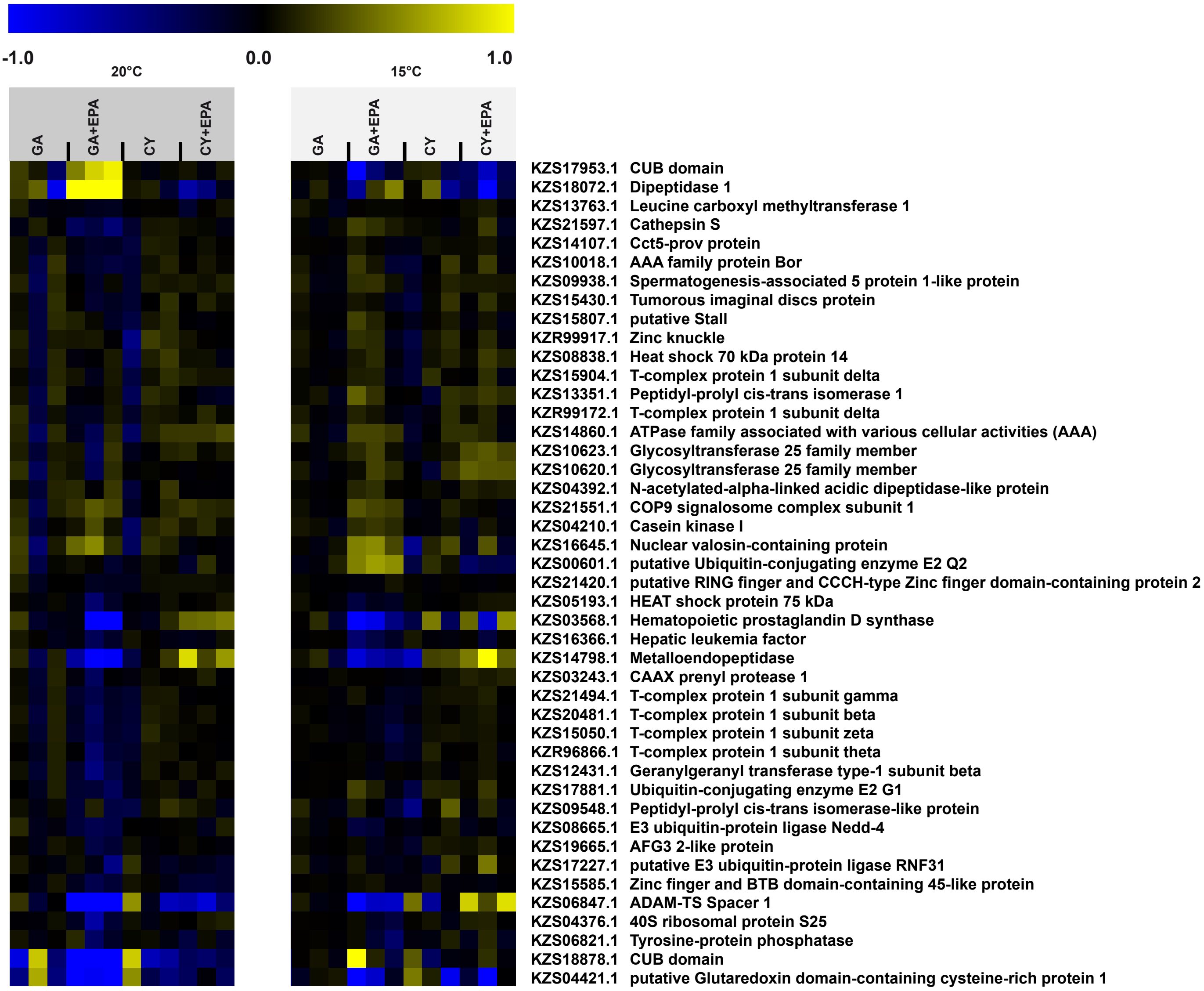

Cellular processes and signalling

Category T - Signal transduction mechanisms

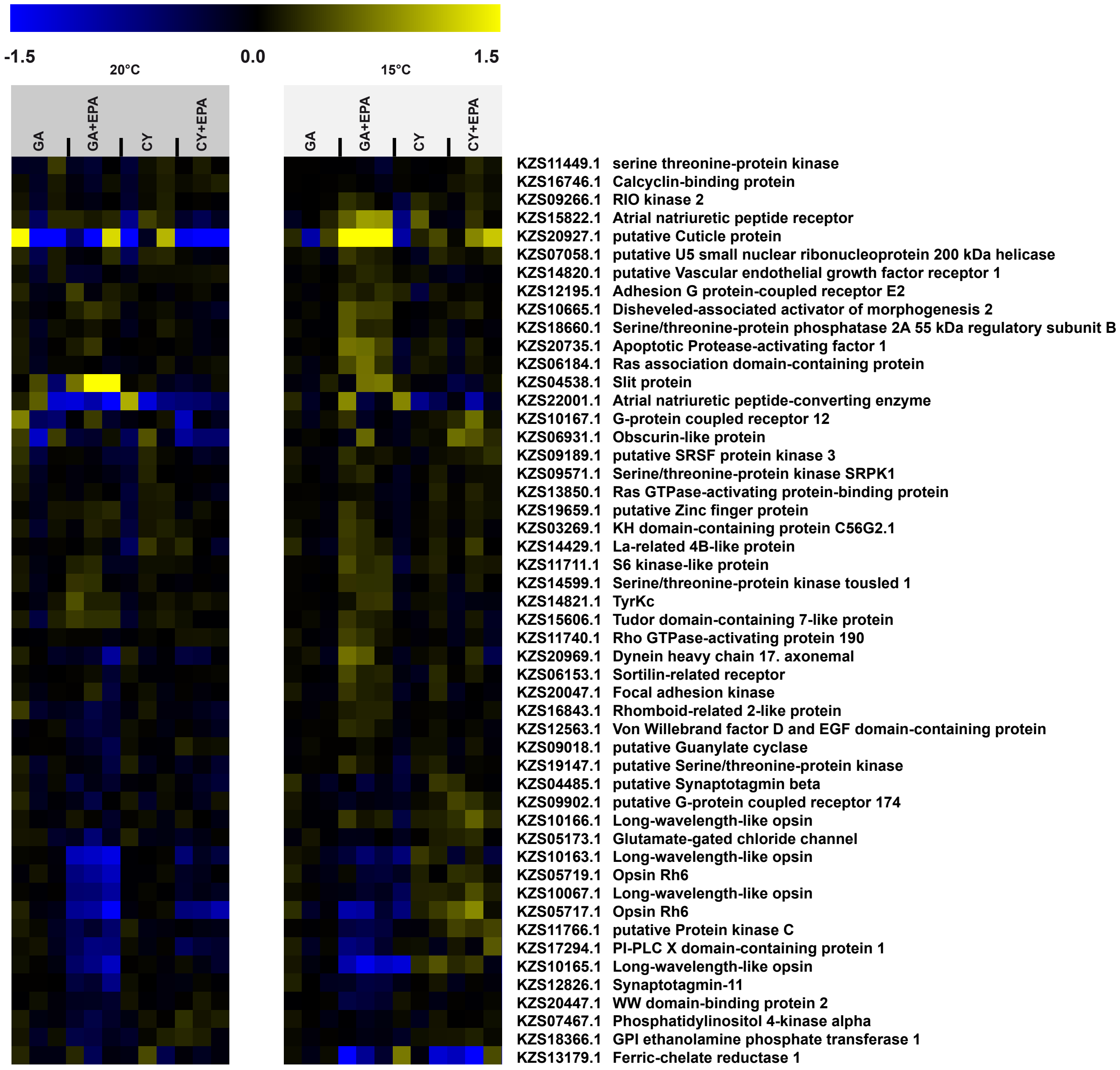

Cellular processes and signalling

Category U - Intracellular trafficking, secretion, and vesicular transport

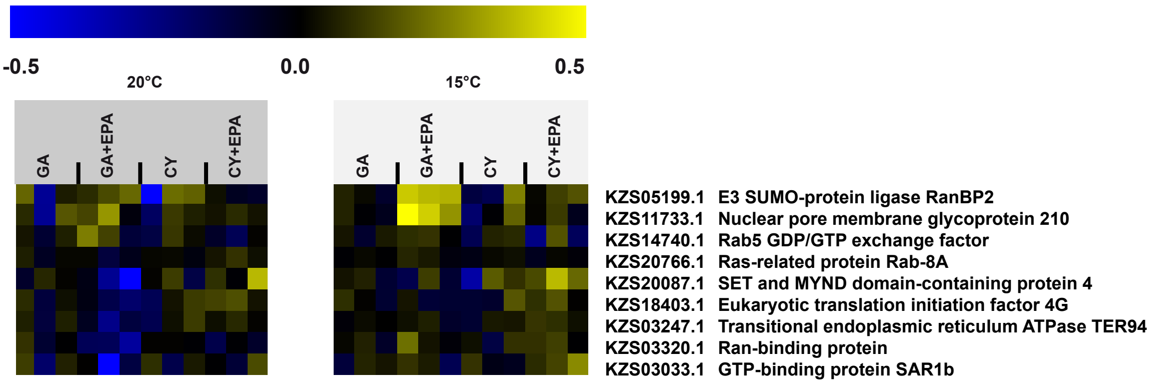

Cellular processes and signalling

Category Z - Cytoskeleton

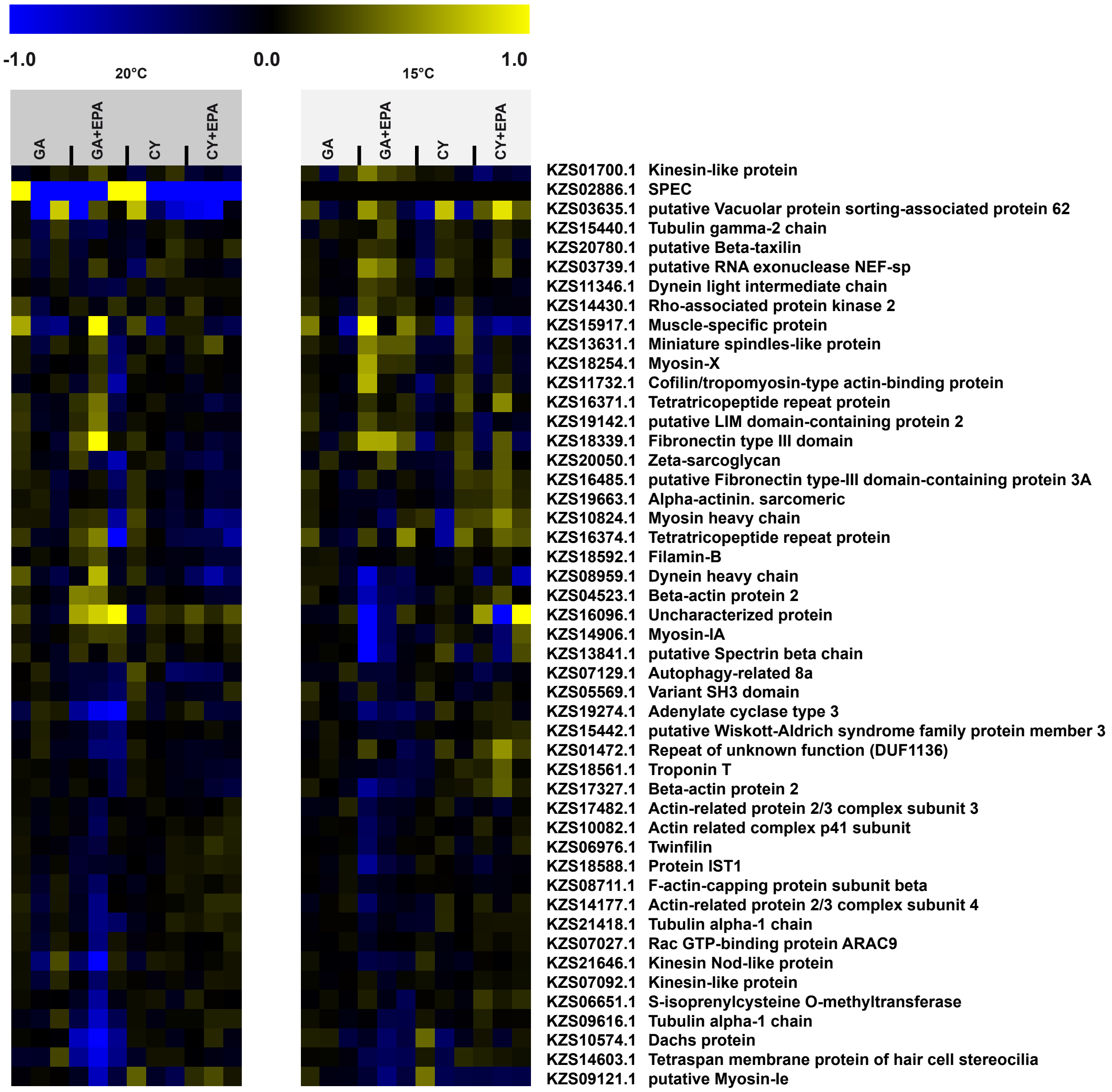

Metabolism

Category E - Amino acid transport and metabolism

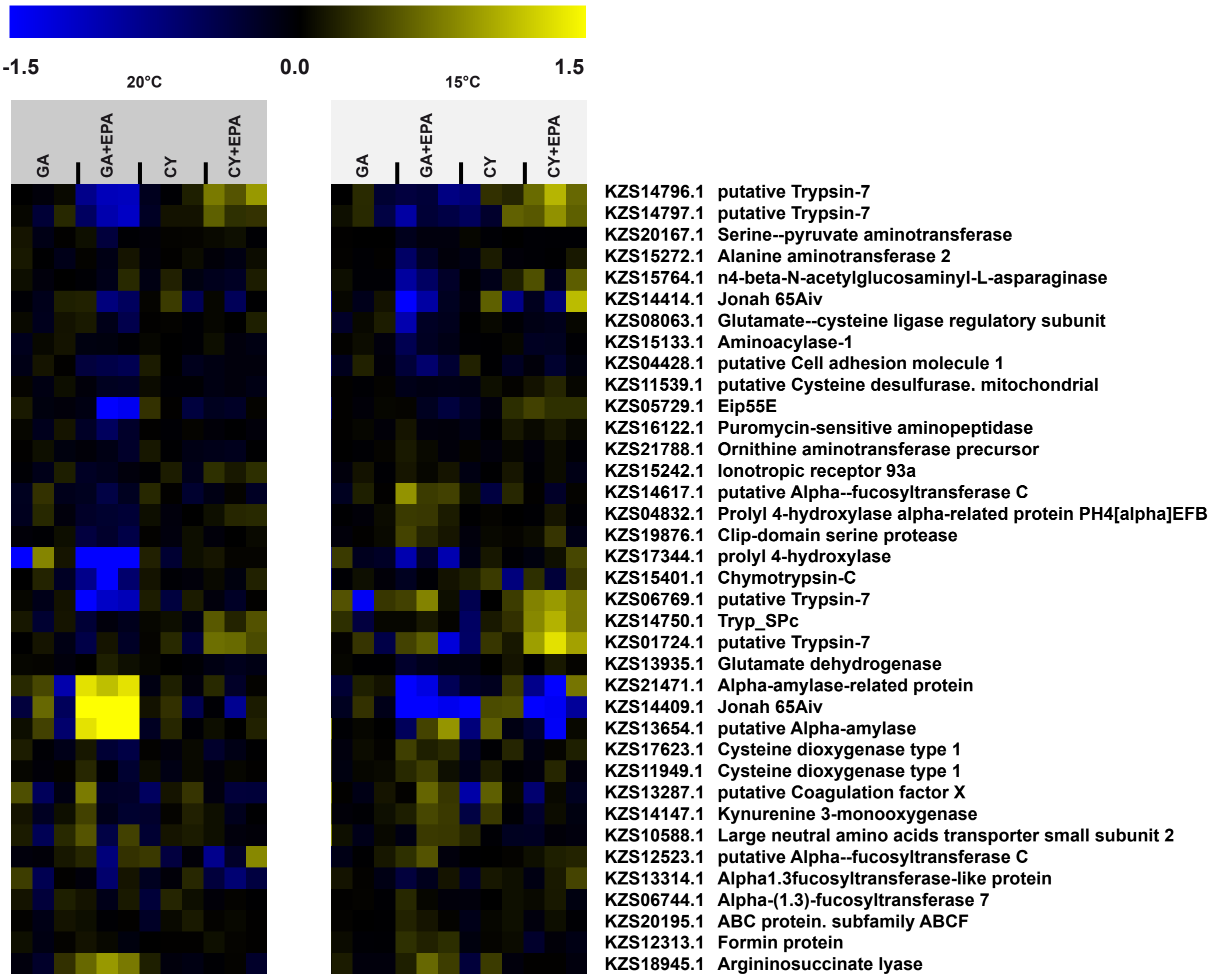

Metabolism

Category G - Carbohydrate transport and metabolism

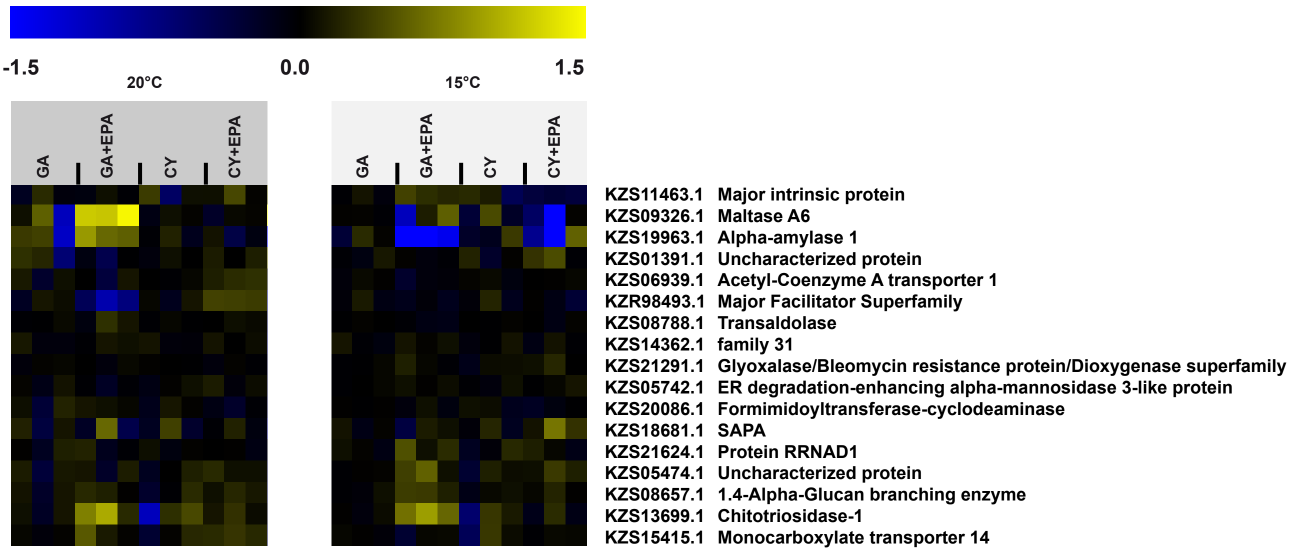

Metabolism

Category I - Lipid transport and metabolism

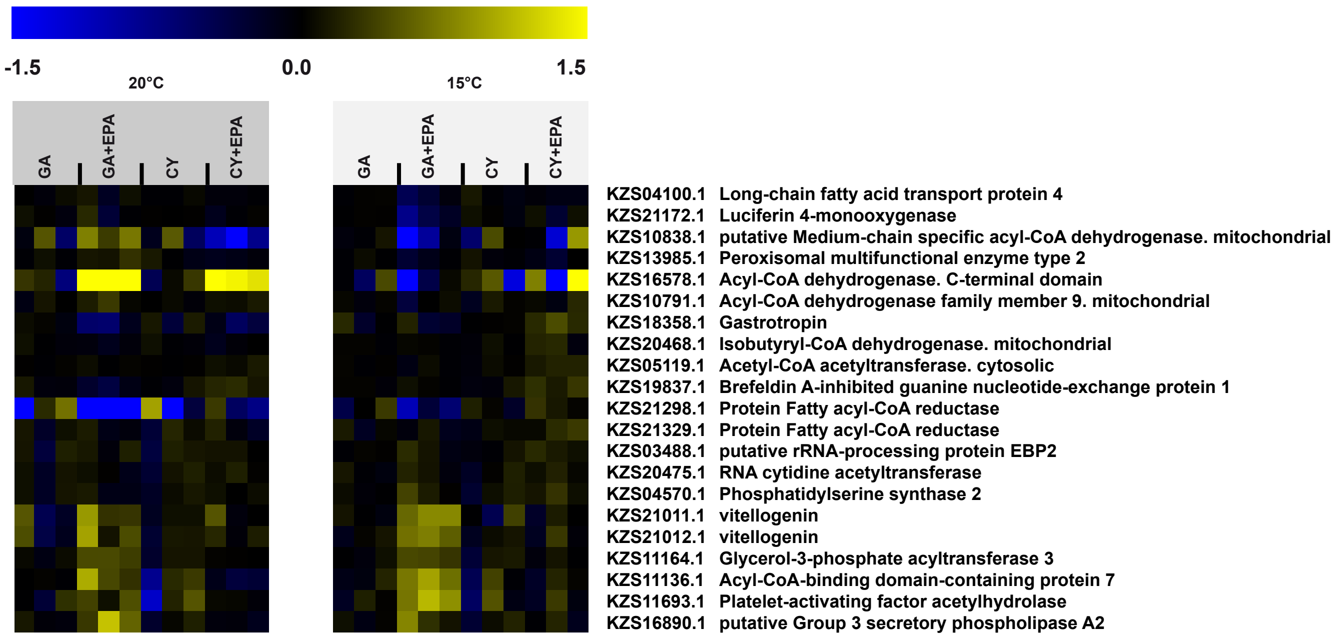

Metabolism

Category P - Inorganic ion transport and metabolism

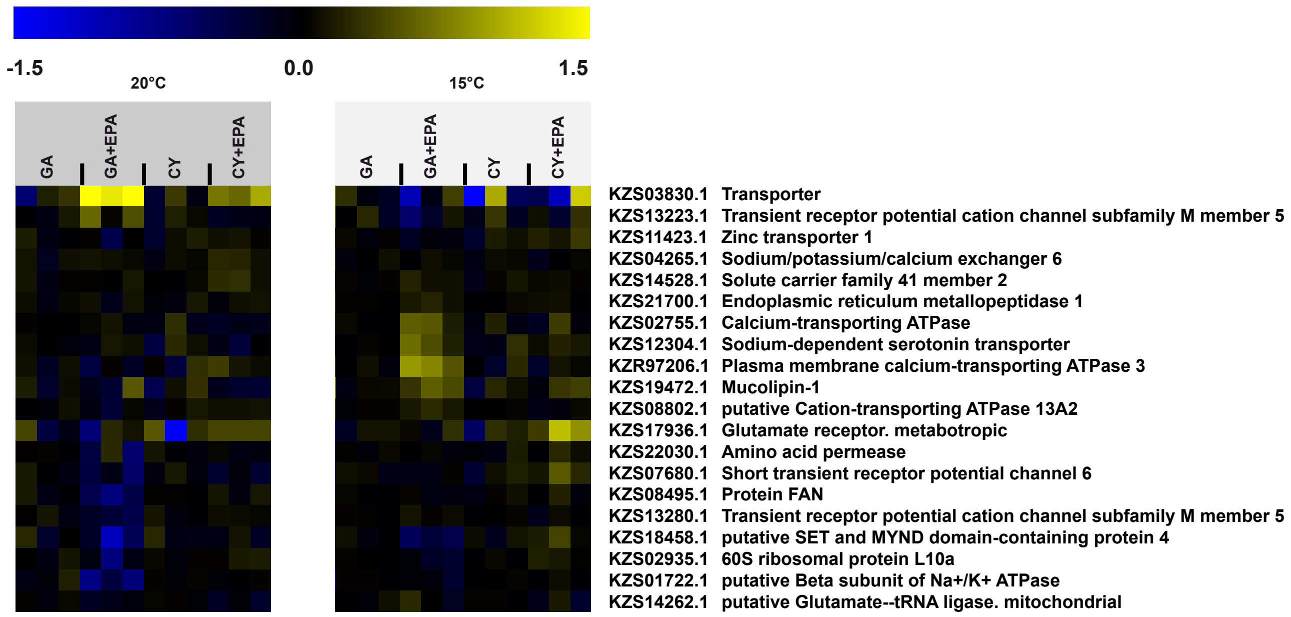

Metabolism

Category Q - Secondary metabolites biosynthesis, transport and catabolism

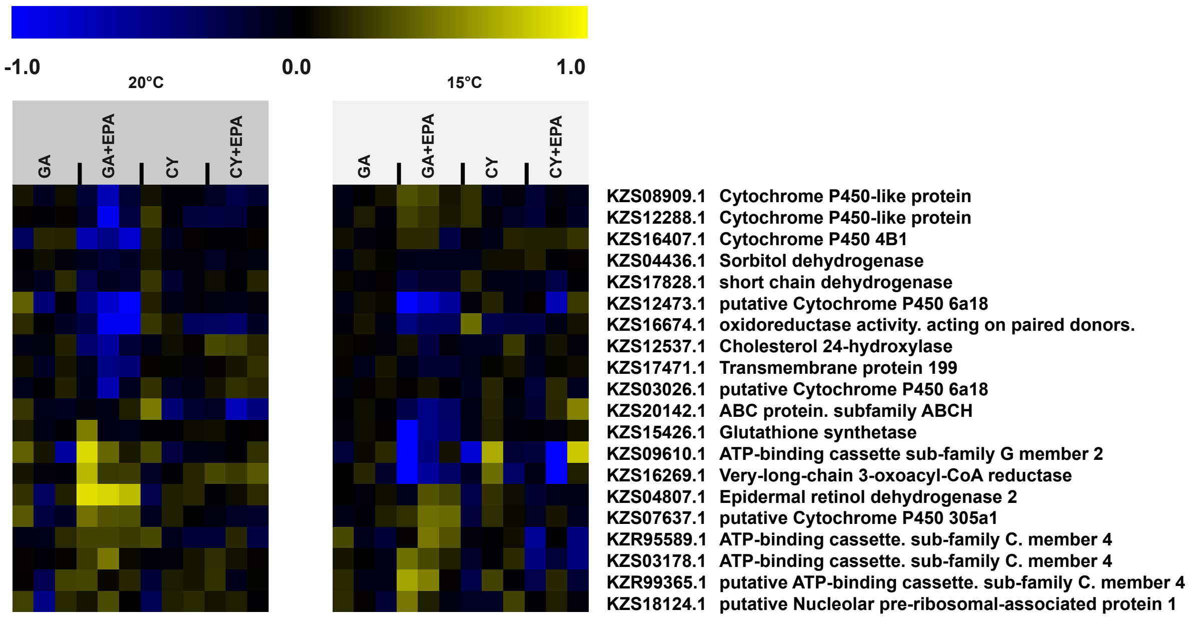

Poorly characterized genes
Category R - General function prediction only in artNOG

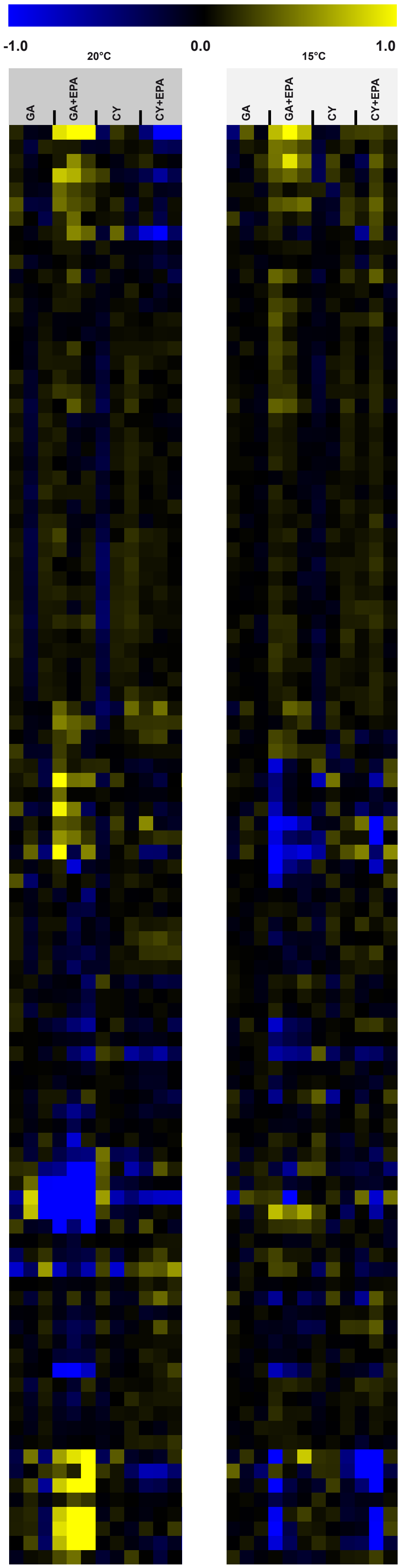

KZS13750.1 Uncharacterized protein
KZS01323.1 Proline-rich nuclear receptor coactivator 2
KZS06270.1 Uncharacterized protein
KZS02956.1 FR47-like protein
KZS09352.1 MAM domain-containing glycosylphosphatidylinositol anchor protein 1
KZS19696.1 Uncharacterized protein
KZS04851.1 Uncharacterized protein
KZS20494.1 Acetyltransferase (GNAT) family
KZS09134.1 SecA DEAD-like domain
KZS19636.1 CD80-like C2-set immunoglobulin domain
KZS13607.1 multiple C2 domain and transmembrane region protein
KZS14485.1 putative Integrator 12
KZS06218.1 Low-density lipoprotein receptor domain class A
KZS11176.1 G patch domain-containing protein 4
KZS21460.1 putative Zinc finger protein 652
KZS14877.1 DNA-binding protein K10
KZS04377.1 WD repeat-containing protein 43
KZS15023.1 putative RNA-binding protein 19
KZS19552.1 Nucleolar GTP-binding protein 2
KZS11051.1 putative Cytoplasmic tRNA 2-thiolation protein 1
KZS03027.1 p21-activated protein kinase-interacting protein 1
KZS08963.1 WD repeat-containing protein 75
KZS07565.1 Ribonuclease Z. mitochondrial
KZS14849.1 putative Eukaryotic translation initiation factor 4B
KZS16258.1 putative Small glutamine-rich tetratricopeptide repeat-containing protein alpha
KZS12926.1 Zinc finger protein RP-8
KZS17100.1 Large subunit GTPase 1
KZS16503.1 Polyribonucleotide nucleotidyltransferase 1. mitochondrial
KZS12314.1 No child left behind
KZS06756.1 WD repeat-containing protein 55
KZS18808.1 putative MKI67 FHA domain-interacting nucleolar phosphoprotein
KZS11276.1 U3 small nucleolar RNA-associated protein 4
KZS18504.1 Protein Peter pan
KZS03715.1 Guanine nucleotide-binding protein 3
KZS15357.1 U3 small nucleolar RNA-associated protein 18
KZS16836.1 U3 small nucleolar RNA-associated protein 6
KZS08720.1 tRNA-dihydrouridine(47) synthase (NAD(P)(+))
KZS12218.1 WD repeat-containing protein 74
KZS07188.1 RNA recognition motif. (a.k.a. RRM. RBD. or RNP domain)
KZS20844.1 ATP-binding cassette sub-family F member 2
KZS22053.1 putative Neuroparsin
KZS12025.1 Delta(24)-sterol reductase
KZR98531.1 putative Multidrug resistance protein 2 atp-binding cassette protein c
KZS13520.1 SecA DEAD-like domain
KZS10128.1 SecA DEAD-like domain
KZS15812.1 Uncharacterized protein
KZS20464.1 Prostaglandin reductase 2
KZS03322.1 Alcohol dehydrogenase A
KZS13190.1 putative Secreted protein
KZS01680.1 Retinol dehydrogenase 12
KZS09623.1 putative Carboxypeptidase B
KZS16634.1 Sulfotransferase 1C2
KZS13197.1 Lactoperoxidase
KZS10376.1 putative Transcription factor A. mitochondrial
KZS03032.1 Iron-sulfur cluster assembly enzyme ISCU
KZS11759.1 Store-operated calcium entry-associated regulatory factor
KZS16160.1 Serine hydrolase
KZS05840.1 Arrestin (or S-antigen). N-terminal domain
KZS12194.1 heterochromatin organization
KZS07804.1 SecA_DEAD
KZS15671.1 Pickpocket 9
KZS14164.1 Uncharacterized protein
KZS20787.1 Corticotropin-releasing factor-binding protein
KZS13253.1 1-acylglycerol-3-phosphate O-acyltransferase ABHD5
KZS02962.1 cAMP-responsive element-binding protein 2
KZS12247.1 Ankyrin repeat
KZS13773.1 Multiple inositol polyphosphate phosphatase 1
KZS03798.1 Uncharacterized protein
KZS04548.1 putative FERM domain-containing protein 4B
KZS01020.1 Beclin-1-like protein
KZS08421.1 Extra macrochaetae-like protein
KZS17958.1 Actin-87e
KZS00239.1 Uncharacterized protein
KZS16703.1 Uncharacterized protein
KZS06293.1 Uncharacterized protein
KZS14408.1 Uncharacterized protein
KZR97907.1 Pol-like protein
KZS16425.1 CDGSH iron-sulfur domain-containing protein 1
KZR99578.1 putative Alkyldihydroxyacetonephosphate synthase
KZS15128.1 chemosensory protein
KZS11070.1 Pantothenate kinase 4
KZR99275.1 pol-like protein
KZS04209.1 Guanine nucleotide binding protein. beta subunit
KZS19290.1 Chorion peroxidase
KZS02858.1 Integrin-alpha FG-GAP repeat-containing protein 2
KZS22002.1 Kinase D-interacting substrate of 220 kDa
KZS18427.1 PTN/MK heparin-binding protein family. C-terminal domain
KZS07671.1 Synaptonemal complex protein SC65
KZS08031.1 putative 18S rRNA (guanine-N(7))-methyltransferase
KZS19807.1 Zinc finger protein 257
KZS20340.1 putative Prip interacting protein. pimt
KZS17291.1 Hippocampus abundant transcript 1 protein
KZS18837.1 metal ion binding
KZS04989.1 Carboxylic ester hydrolase
KZS04109.1 Carboxylic ester hydrolase
KZS15376.1 putative ETHE1 protein
KZS15599.1 Peritrophic matrix-like protein
KZS12186.1 Calcium-activated chloride channel regulator 2
KZS16356.1 Uncharacterized protein
KZS20133.1 Cuticular protein

Poorly characterized genes
Category S - Functions unknown in artNOG

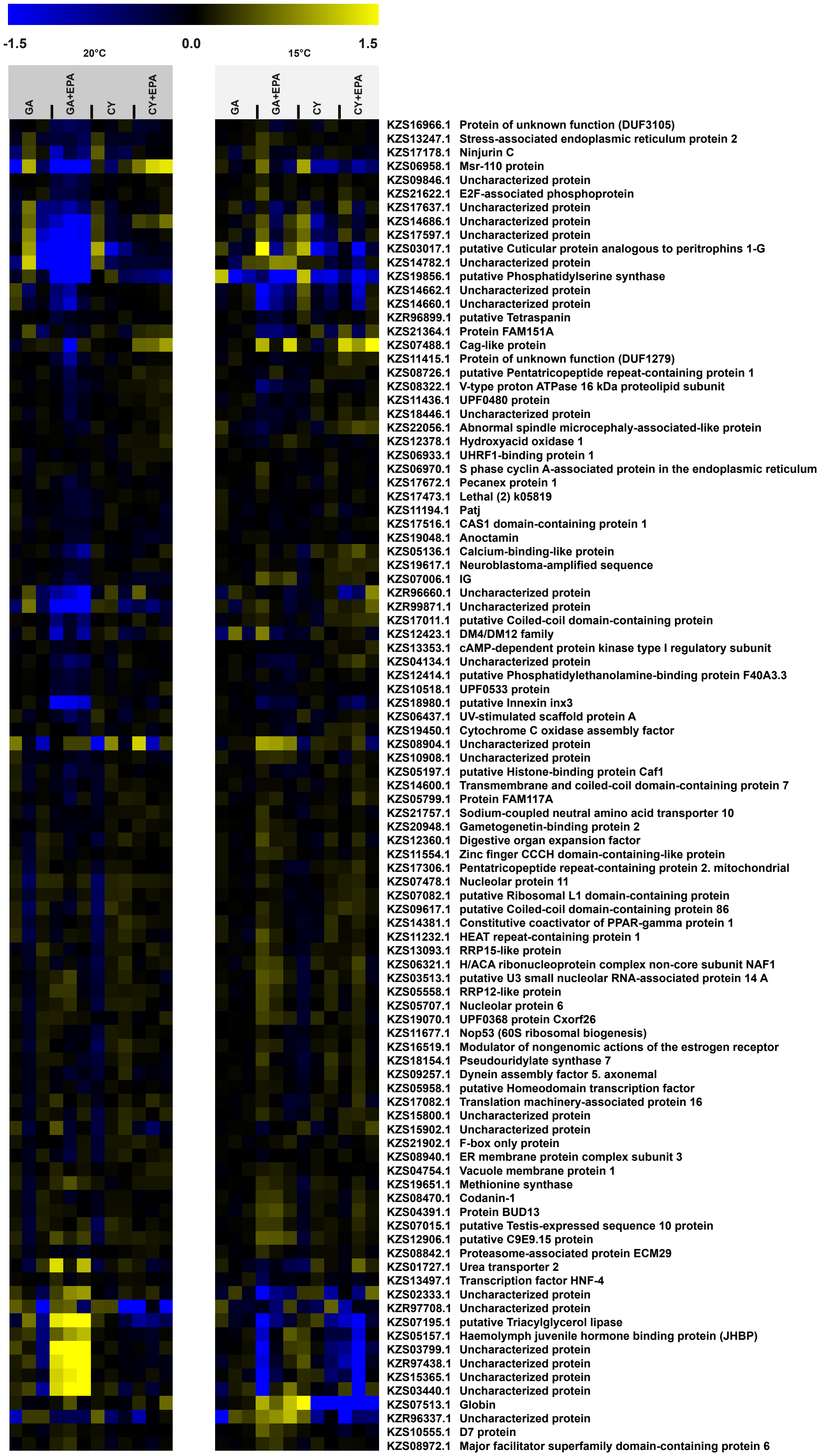

Poorly characterized genes

Category X - No result in artNOG database

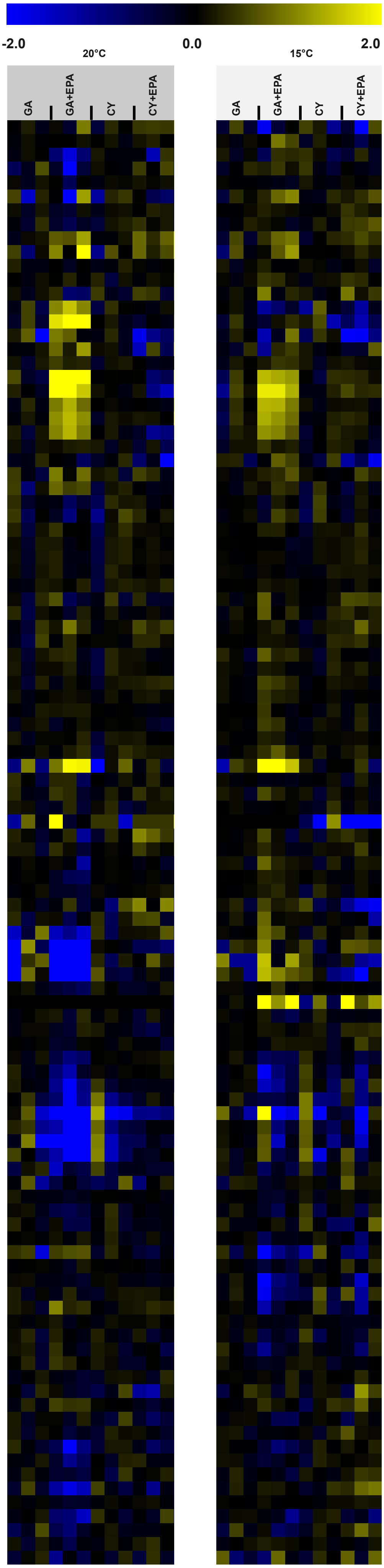

KZS14457.1 Uncharacterized protein
KZS07585.1 Uncharacterized protein
KZS15325.1 Uncharacterized protein
KZS14531.1 Uncharacterized protein
KZR96473.1 Uncharacterized protein
KZS19471.1 Uncharacterized protein
KZS15394.1 Uncharacterized protein
KZS12204.1 Uncharacterized protein
KZS15416.1 Uncharacterized protein
KZS15418.1 Uncharacterized protein
KZS11702.1 Uncharacterized protein
KZS17635.1 Uncharacterized protein
KZS20751.1 Uncharacterized protein
KZS05842.1 Uncharacterized protein
KZS07597.1 Uncharacterized protein
KZS10753.1 Endo-beta-1.4-mannanase
KZR96719.1 Uncharacterized protein
KZS18417.1 putative Transcriptional factor muscle segment homeobox protein
KZR99949.1 Uncharacterized protein
KZR99018.1 Uncharacterized protein
KZR97320.1 Uncharacterized protein
KZS08524.1 Uncharacterized protein
KZS13749.1 Uncharacterized protein
KZS12522.1 Uncharacterized protein
KZR99211.1 Uncharacterized protein
KZS00326.1 Uncharacterized protein
KZS06947.1 Uncharacterized protein
KZS16940.1 Uncharacterized protein
KZS04613.1 Uncharacterized protein
KZS11625.1 Uncharacterized protein
KZR97720.1 Uncharacterized protein
KZS11619.1 Uncharacterized protein
KZS04831.1 Uncharacterized protein
KZS00111.1 Uncharacterized protein
KZS12645.1 Uncharacterized protein
KZS13200.1 Uncharacterized protein
KZS11361.1 Uncharacterized protein
KZR99393.1 Uncharacterized protein
KZS04336.1 Uncharacterized protein
KZS18609.1 Zinc finger cchc domain containing-like protein
KZS04335.1 Uncharacterized protein
KZS09284.1 Uncharacterized protein
KZS05640.1 Uncharacterized protein
KZS14866.1 Uncharacterized protein
KZR98714.1 Uncharacterized protein
KZS19990.1 Uncharacterized protein
KZS13662.1 Uncharacterized protein
KZS11411.1 Uncharacterized protein
KZS06154.1 putative Zinc finger protein 618
KZS11477.1 Uncharacterized protein
KZS03711.1 Uncharacterized protein
KZS06252.1 Uncharacterized protein
KZS04852.1 Uncharacterized protein
KZS17204.1 Uncharacterized protein
KZS11462.1 putative C1q and tumor necrosis factor-related protein 2
KZS16553.1 Uncharacterized protein
KZS10216.1 Uncharacterized protein
KZS14847.1 Uncharacterized protein
KZS19418.1 Uncharacterized protein
KZS04280.1 Uncharacterized protein
KZS19119.1 Uncharacterized protein
KZS19118.1 Uncharacterized protein
KZS08984.1 Uncharacterized protein
KZS07160.1 Uncharacterized protein
KZS06787.1 Uncharacterized protein
KZR97941.1 Uncharacterized protein
KZS00236.1 Uncharacterized protein
KZS20280.1 Uncharacterized protein
KZS14661.1 Uncharacterized protein
KZS14468.1 Uncharacterized protein
KZS05356.1 Uncharacterized protein
KZS10891.1 Uncharacterized protein
KZS17021.1 Uncharacterized protein
KZS16807.1 Uncharacterized protein
KZS06855.1 Uncharacterized protein
KZS00820.1 Uncharacterized protein
KZS13161.1 Uncharacterized protein
KZS03864.1 Uncharacterized protein
KZS04429.1 Uncharacterized protein
KZS01822.1 Uncharacterized protein
KZS00999.1 Uncharacterized protein
KZS17998.1 Cellobiohydrolase CHBI
KZS12755.1 Uncharacterized protein
KZS12036.1 Uncharacterized protein
KZS12496.1 Uncharacterized protein
KZS21174.1 Vitelline membrane outer layer protein 1
KZS21279.1 Uncharacterized protein
KZS03966.1 Helicase c-terminal domain containing-like protein
KZR96833.1 Uncharacterized protein
KZS01823.1 Uncharacterized protein
KZS21053.1 putative Swi5-dependent recombination DNA repair protein 1
KZS20708.1 Uncharacterized protein
KZR99922.1 Uncharacterized protein
KZS15296.1 Uncharacterized protein
KZS14554.1 Uncharacterized protein
KZR96069.1 Uncharacterized protein
KZS07662.1 Uncharacterized protein
KZS13726.1 Uncharacterized protein
KZS16599.1 Uncharacterized protein
KZS10918.1 Uncharacterized protein
KZS19207.1 Uncharacterized protein
KZR96250.1 Uncharacterized protein
KZS05825.1 Uncharacterized protein
KZR97757.1 Uncharacterized protein
