# Supplementary material for: Transcriptome sequencing of a keystone aquatic herbivore yields insights on the temperature-dependent metabolism of essential lipids
Source: BMC Genomics. 2019 Nov 21;20:894. doi: 10.1186/s12864-019-6268-y (PMC6873670; doi:10.1186/s12864-019-6268-y)
Supplement: Supplementary file 3 — Additional file 3. EPA significant gene expression profiles and combined effects. [file 12864_2019_6268_MOESM3_ESM.pdf]

EPA - significant genes

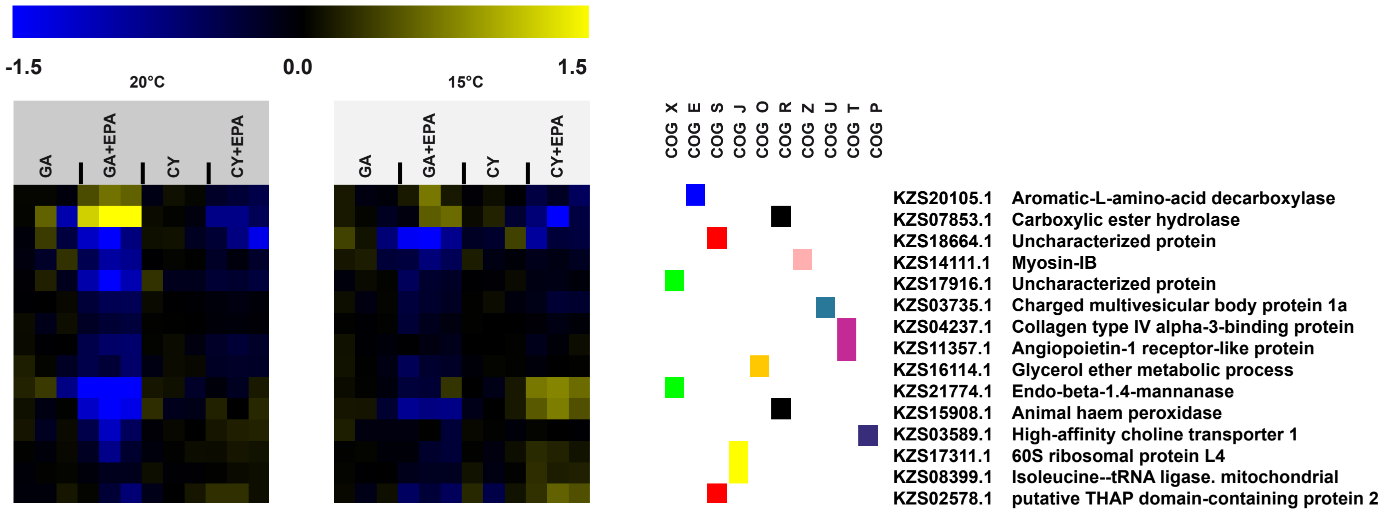

Combined effects of EPA and temperature

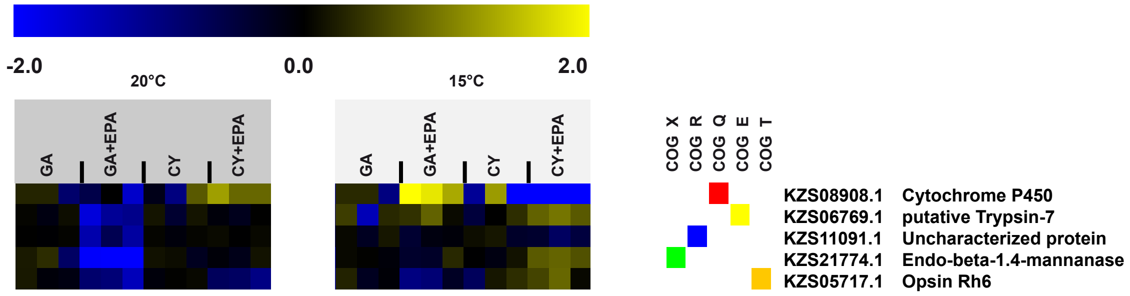

Abbreviations

[E] Amino acid transport and metabolism
[J] Translation, ribosomal structure and biogenesis
[O] Posttranslational modification, protein turnover & chaperones
[P] Inorganic ion transport and metabolism
[Q] Secondary metabolites biosynthesis, transport and catabolism
[R] General function prediction only in artNOG
[S] Function unknown in artNOG
[T] Signal transduction mechanisms
[U] Intracellular trafficking, secretion, and vesicular transport
[X] No result in artNOG
[Z] Cytoskeleton
