# Supplementary material for: Transcriptome sequencing of a keystone aquatic herbivore yields insights on the temperature-dependent metabolism of essential lipids
Source: BMC Genomics. 2019 Nov 21;20:894. doi: 10.1186/s12864-019-6268-y (PMC6873670; doi:10.1186/s12864-019-6268-y)
Supplement: Supplementary file 4 — Additional file 4. Expression profiles of “poorly characterized” genes responsive to temperature, EPA and combined effects. [file 12864_2019_6268_MOESM4_ESM.pdf]

Poorly characterized genes responsive to temperature
Category R - General function prediction only

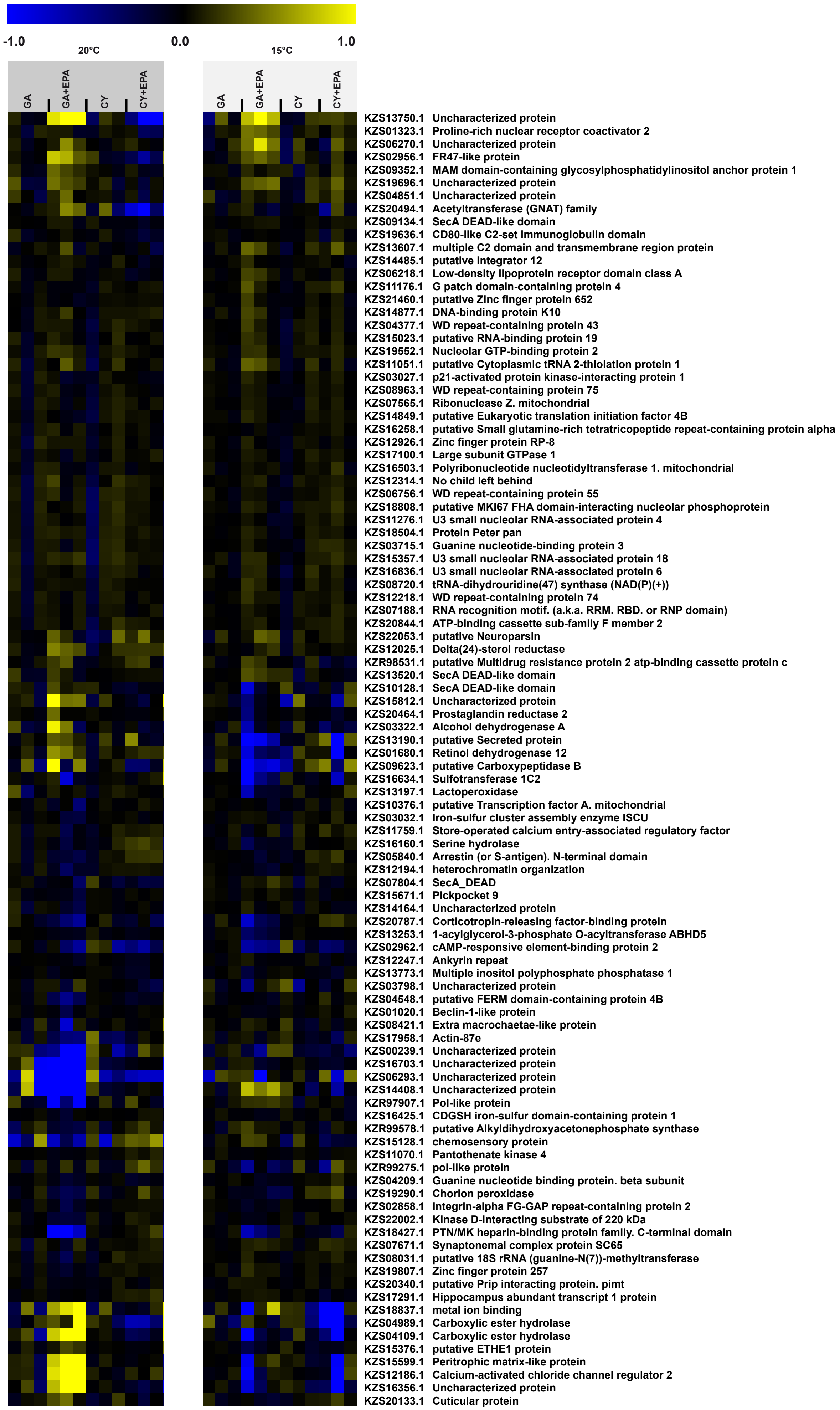

Poorly characterized genes responsive to temperature
Category S - Functions unknown

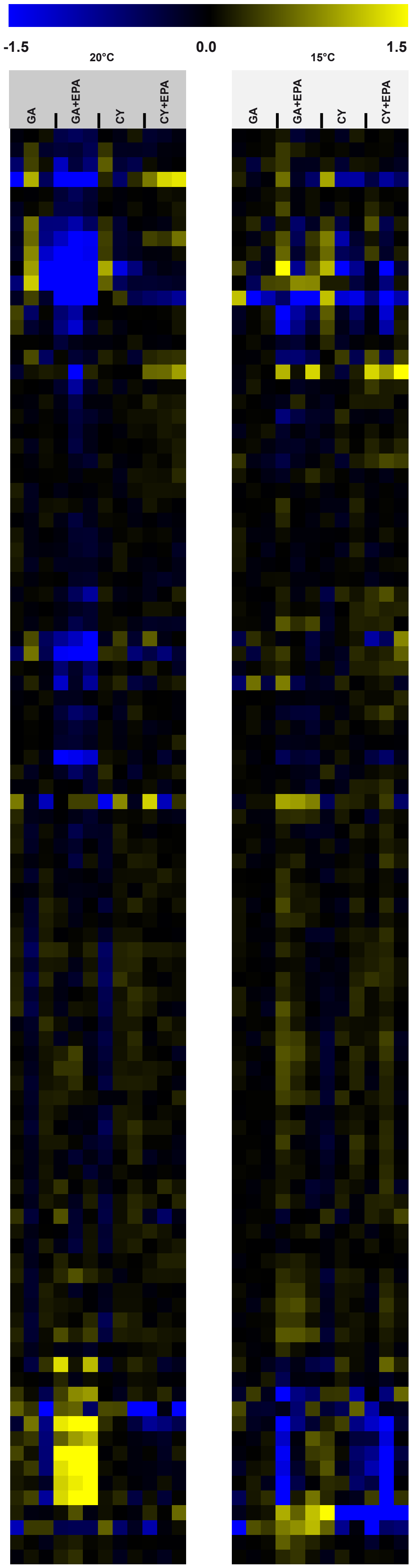

KZS16966.1 Protein of unknown function (DUF3105)
KZS13247.1 Stress-associated endoplasmic reticulum protein 2
KZS17178.1 Ninjurin C
KZS06958.1 Msr-110 protein
KZS09846.1 Uncharacterized protein
KZS21622.1 E2F-associated phosphoprotein
KZS17637.1 Uncharacterized protein
KZS14686.1 Uncharacterized protein
KZS17597.1 Uncharacterized protein
KZS03017.1 putative Cuticular protein analogous to peritrophins 1-G
KZS14782.1 Uncharacterized protein
KZS19856.1 putative Phosphatidylserine synthase
KZS14662.1 Uncharacterized protein
KZS14660.1 Uncharacterized protein
KZR96899.1 putative Tetraspanin
KZS21364.1 Protein FAM151A
KZS07488.1 Cag-like protein
KZS11415.1 Protein of unknown function (DUF1279)
KZS08726.1 putative Pentatricopeptide repeat-containing protein 1
KZS08322.1 V-type proton ATPase 16 kDa proteolipid subunit
KZS11436.1 UPF0480 protein
KZS18446.1 Uncharacterized protein
KZS22056.1 Abnormal spindle microcephaly-associated-like protein
KZS12378.1 Hydroxyacid oxidase 1
KZS06933.1 UHRF1-binding protein 1
KZS06970.1 S phase cyclin A-associated protein in the endoplasmic reticulum
KZS17672.1 Pecanex protein 1
KZS17473.1 Lethal (2) k05819
KZS11194.1 Patj
KZS17516.1 CAS1 domain-containing protein 1
KZS19048.1 Anoctamin
KZS05136.1 Calcium-binding-like protein
KZS19617.1 Neuroblastoma-amplified sequence
KZS07006.1 IG
KZR96660.1 Uncharacterized protein
KZR99871.1 Uncharacterized protein
KZS17011.1 putative Coiled-coil domain-containing protein
KZS12423.1 DM4/DM12 family
KZS13353.1 cAMP-dependent protein kinase type I regulatory subunit
KZS04134.1 Uncharacterized protein
KZS12414.1 putative Phosphatidylethanolamine-binding protein F40A3.3
KZS10518.1 UPF0533 protein
KZS18980.1 putative Innexin inx3
KZS06437.1 UV-stimulated scaffold protein A
KZS19450.1 Cytochrome C oxidase assembly factor
KZS08904.1 Uncharacterized protein
KZS10908.1 Uncharacterized protein
KZS05197.1 putative Histone-binding protein Caf1
KZS14600.1 Transmembrane and coiled-coil domain-containing protein 7
KZS05799.1 Protein FAM117A
KZS21757.1 Sodium-coupled neutral amino acid transporter 10
KZS20948.1 Gametogenetin-binding protein 2
KZS12360.1 Digestive organ expansion factor
KZS11554.1 Zinc finger CCCH domain-containing-like protein
KZS17306.1 Pentatricopeptide repeat-containing protein 2. mitochondrial
KZS07478.1 Nucleolar protein 11
KZS07082.1 putative Ribosomal L1 domain-containing protein
KZS09617.1 putative Coiled-coil domain-containing protein 86
KZS14381.1 Constitutive coactivator of PPAR-gamma protein 1
KZS11232.1 HEAT repeat-containing protein 1
KZS13093.1 RRP15-like protein
KZS06321.1 H/ACA ribonucleoprotein complex non-core subunit NAF1
KZS03513.1 putative U3 small nucleolar RNA-associated protein 14 A
KZS05558.1 RRP12-like protein
KZS05707.1 Nucleolar protein 6
KZS19070.1 UPF0368 protein Cxorf26
KZS11677.1 Nop53 (60S ribosomal biogenesis)
KZS16519.1 Modulator of nongenomic actions of the estrogen receptor
KZS18154.1 Pseudouridylate synthase 7
KZS09257.1 Dynein assembly factor 5. axonemal
KZS05958.1 putative Homeodomain transcription factor
KZS17082.1 Translation machinery-associated protein 16
KZS15800.1 Uncharacterized protein
KZS15902.1 Uncharacterized protein
KZS21902.1 F-box only protein
KZS08940.1 ER membrane protein complex subunit 3
KZS04754.1 Vacuole membrane protein 1
KZS19651.1 Methionine synthase
KZS08470.1 Codanin-1
KZS04391.1 Protein BUD13
KZS07015.1 putative Testis-expressed sequence 10 protein
KZS12906.1 putative C9E9.15 protein
KZS08842.1 Proteasome-associated protein ECM29
KZS01727.1 Urea transporter 2
KZS13497.1 Transcription factor HNF-4
KZS02333.1 Uncharacterized protein
KZR97708.1 Uncharacterized protein
KZS07195.1 putative Triacylglycerol lipase
KZS05157.1 Haemolymph juvenile hormone binding protein (JHBP)
KZS03799.1 Uncharacterized protein
KZR97438.1 Uncharacterized protein
KZS15365.1 Uncharacterized protein
KZS03440.1 Uncharacterized protein
KZS07513.1 Globin
KZR96337.1 Uncharacterized protein
KZS10555.1 D7 protein
KZS08972.1 Major facilitator superfamily domain-containing protein 6

Poorly characterized genes responsive to temperature
Category X - No result in ArtNOG database

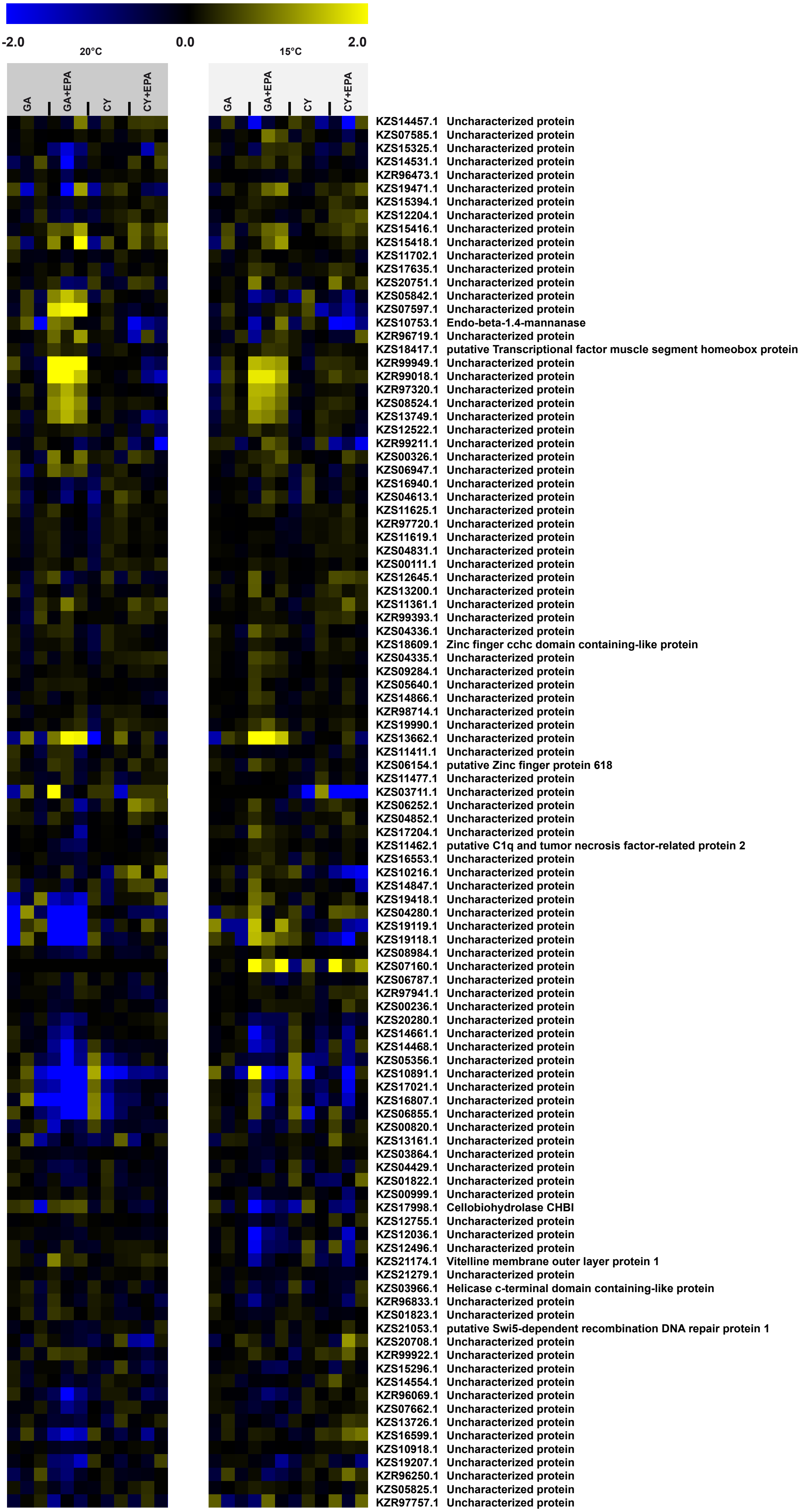

EPA - significant genes and

Combined effects of EPA and temperature

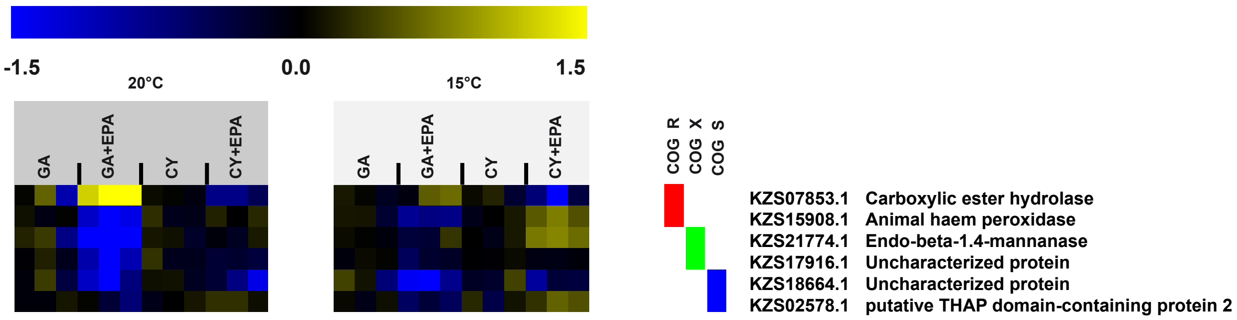

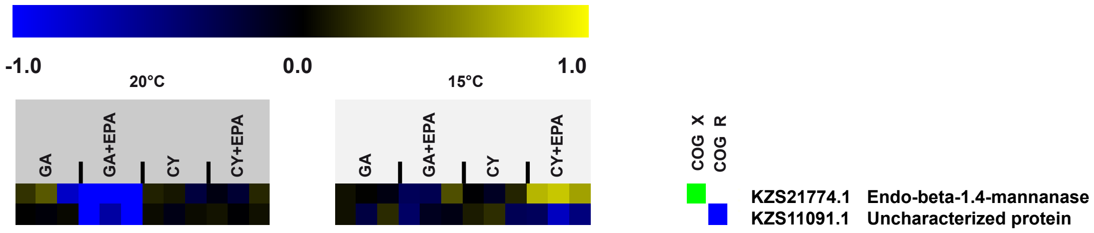

Abbreviations

[R] General function prediction only
[S] Function unknown
[X] No match in artNOG
