# Supplementary material for: Transcriptome sequencing of a keystone aquatic herbivore yields insights on the temperature-dependent metabolism of essential lipids
Source: BMC Genomics. 2019 Nov 21;20:894. doi: 10.1186/s12864-019-6268-y (PMC6873670; doi:10.1186/s12864-019-6268-y)
Supplement: Supplementary file 5 — Additional file 5: Volcano plots of transcripts with LOG2-fold changes > 1 (identified by two-way ANOVA, based on means of triplicates in each group). Respective values were plotted against the adjusted p-values. A respective COG-annotation of single transcripts was included by colour of the data points. Single data points were also labelled with product accession numbers for identification. The underlying data sets were exported as Additional file 6. [file 12864_2019_6268_MOESM5_ESM.pdf]

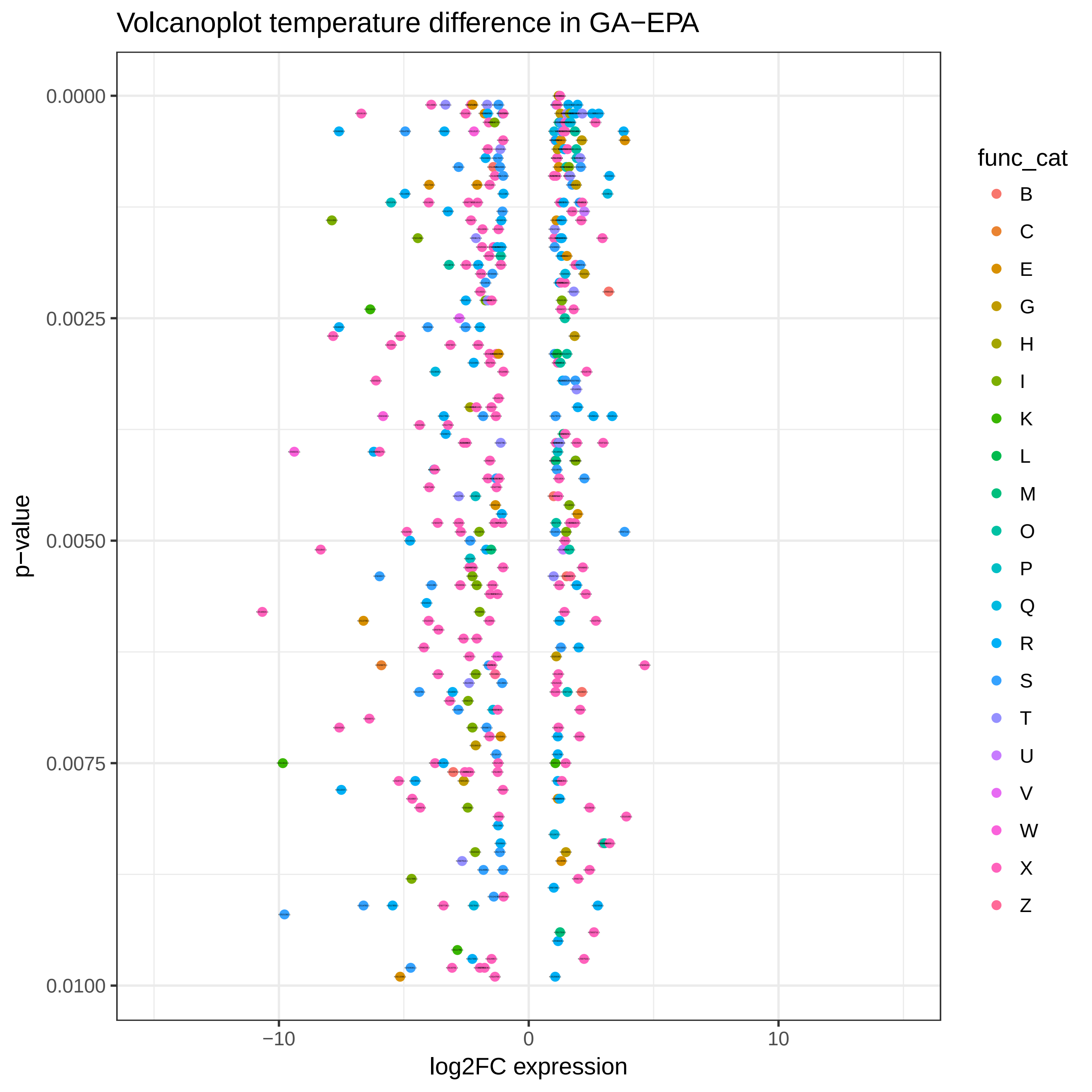

Volcanoplot temperature difference in GA−EPA
p−value
0.0000
0.0025
0.0050
0.0075
0.0100
log2FC expression
−10
0
10
func_cat
B
C
E
G
H
I
K
L
M
O
P
Q
R
S
T
U
V
W
X
Z

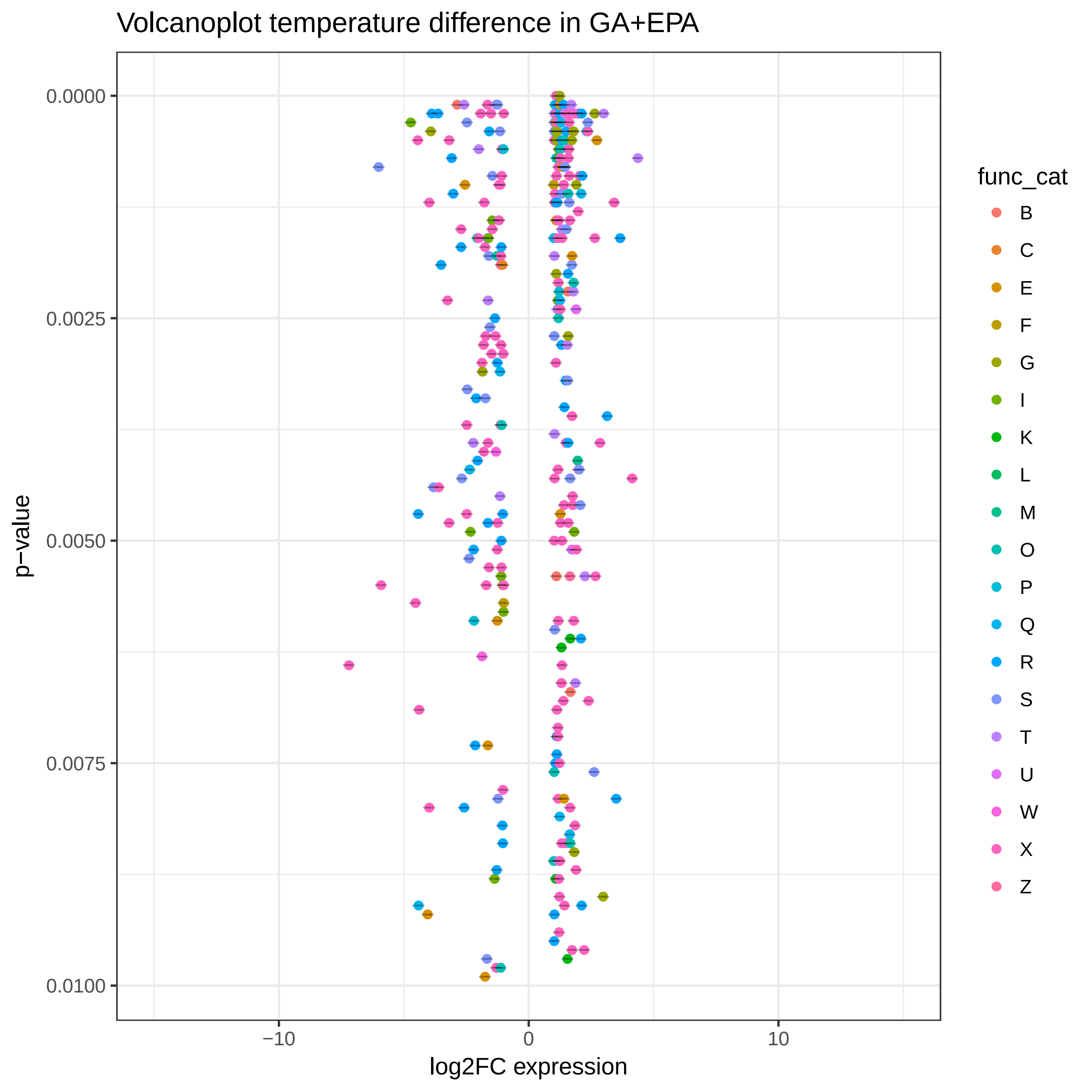
Volcanoplot temperature difference in GA+EPA
p−value
0.0000
0.0025
0.0050
0.0075
0.0100
log2FC expression
−10
0
10
func_cat
B
C
E
F
G
I
K
L
M
O
P
Q
R
S
T
U
W
X
Z

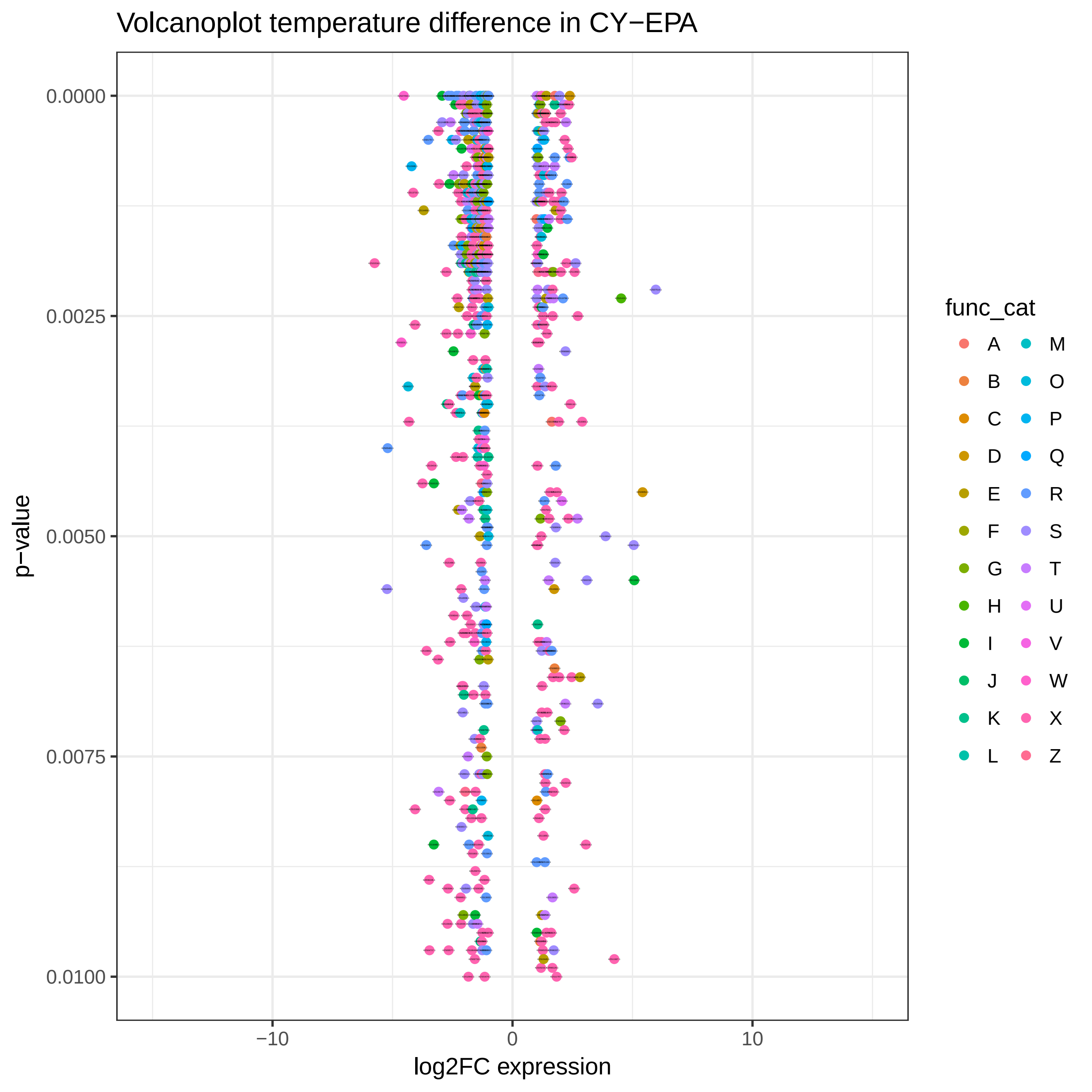

Volcanoplot temperature difference in CY−EPA
p−value
0.0000
0.0025
0.0050
0.0075
0.0100
−10
0
10
log2FC expression
func_cat
A
B
C
D
E
F
G
H
I
J
K
L
M
O
P
Q
R
S
T
U
V
W
X
Z

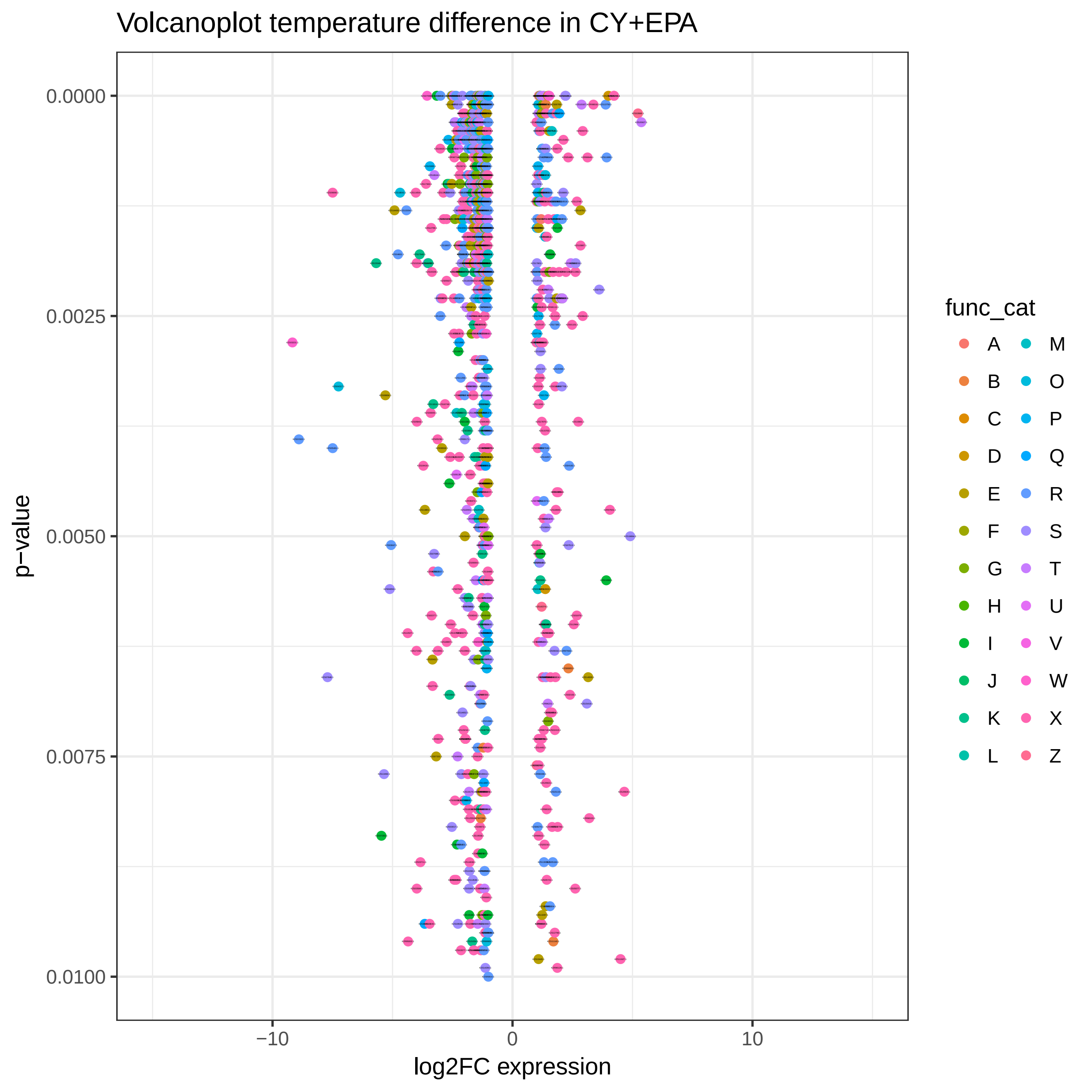
Volcanoplot temperature difference in CY+EPA
p−value
0.0000
0.0025
0.0050
0.0075
0.0100
−10
0
10
log2FC expression
func_cat
A
B
C
D
E
F
G
H
I
J
K
L
M
O
P
Q
R
S
T
U
V
W
X
Z

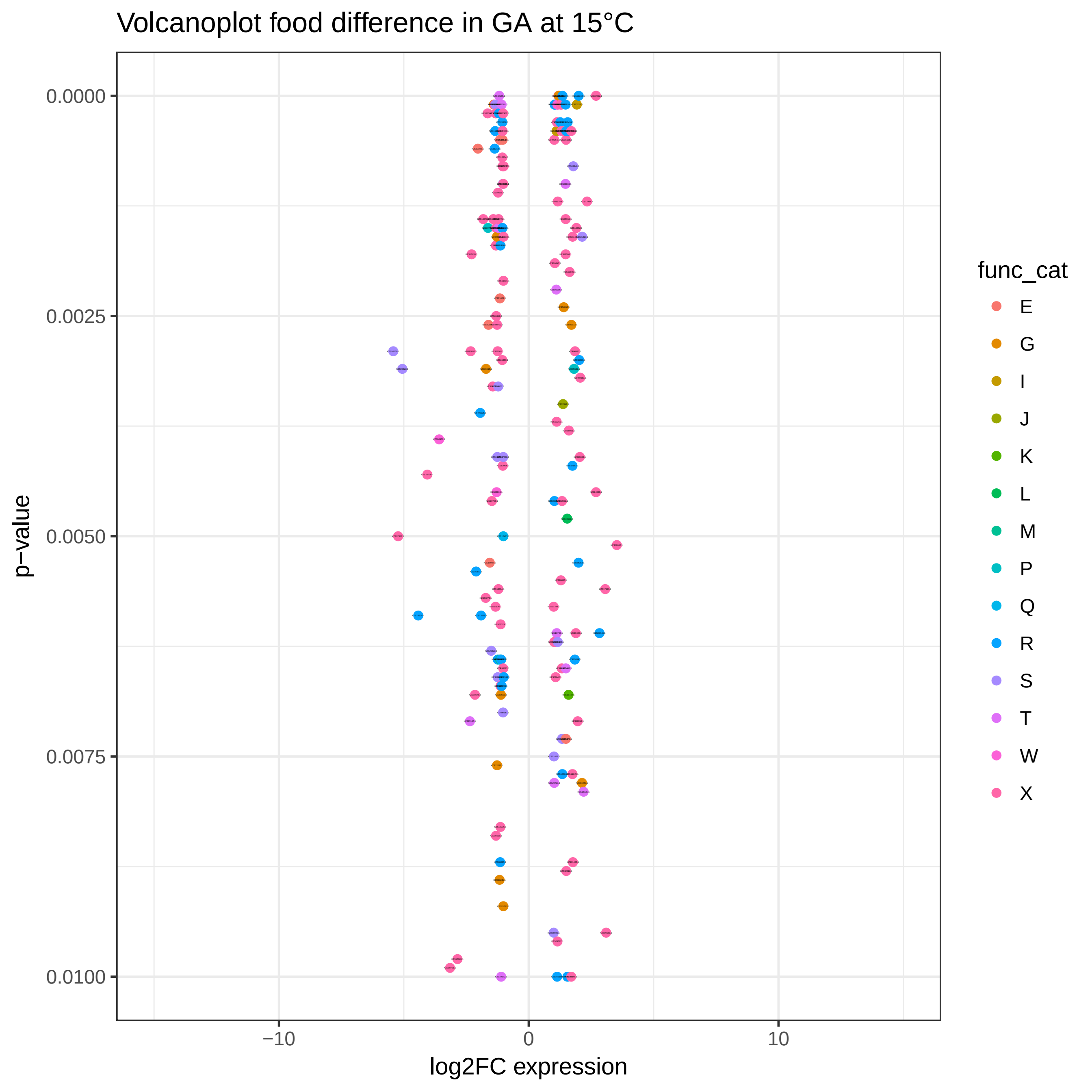

Volcanoplot food difference in GA at 15°C
p−value
0.0000
0.0025
0.0050
0.0075
0.0100
−10
0
10
log2FC expression
func_cat
E
G
I
J
K
L
M
P
Q
R
S
T
W
X

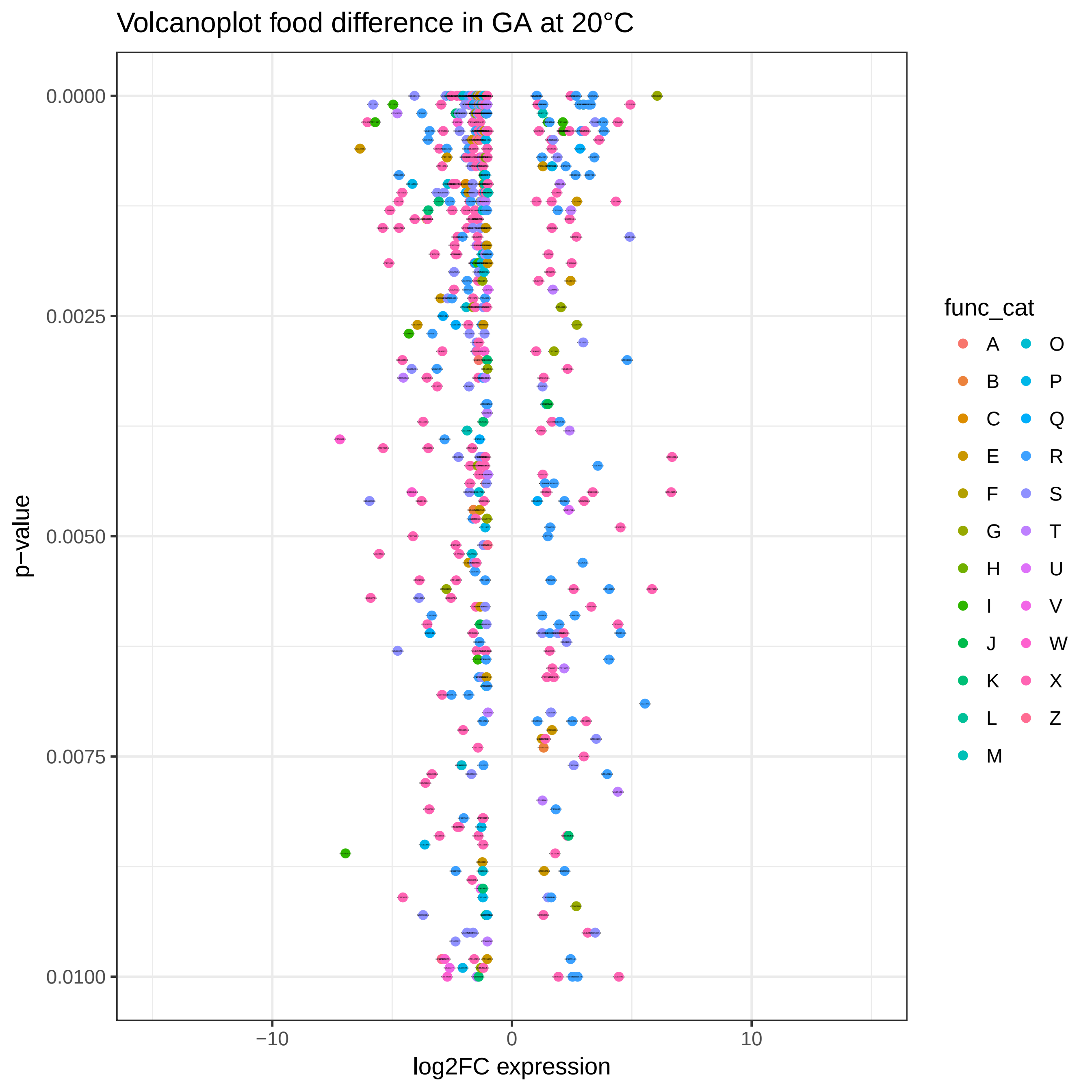

Volcanoplot food difference in GA at 20°C
p−value
0.0000
0.0025
0.0050
0.0075
0.0100
−10
0
10
log2FC expression
func_cat
A
B
C
E
F
G
H
I
J
K
L
M
O
P
Q
R
S
T
U
V
W
X
Z

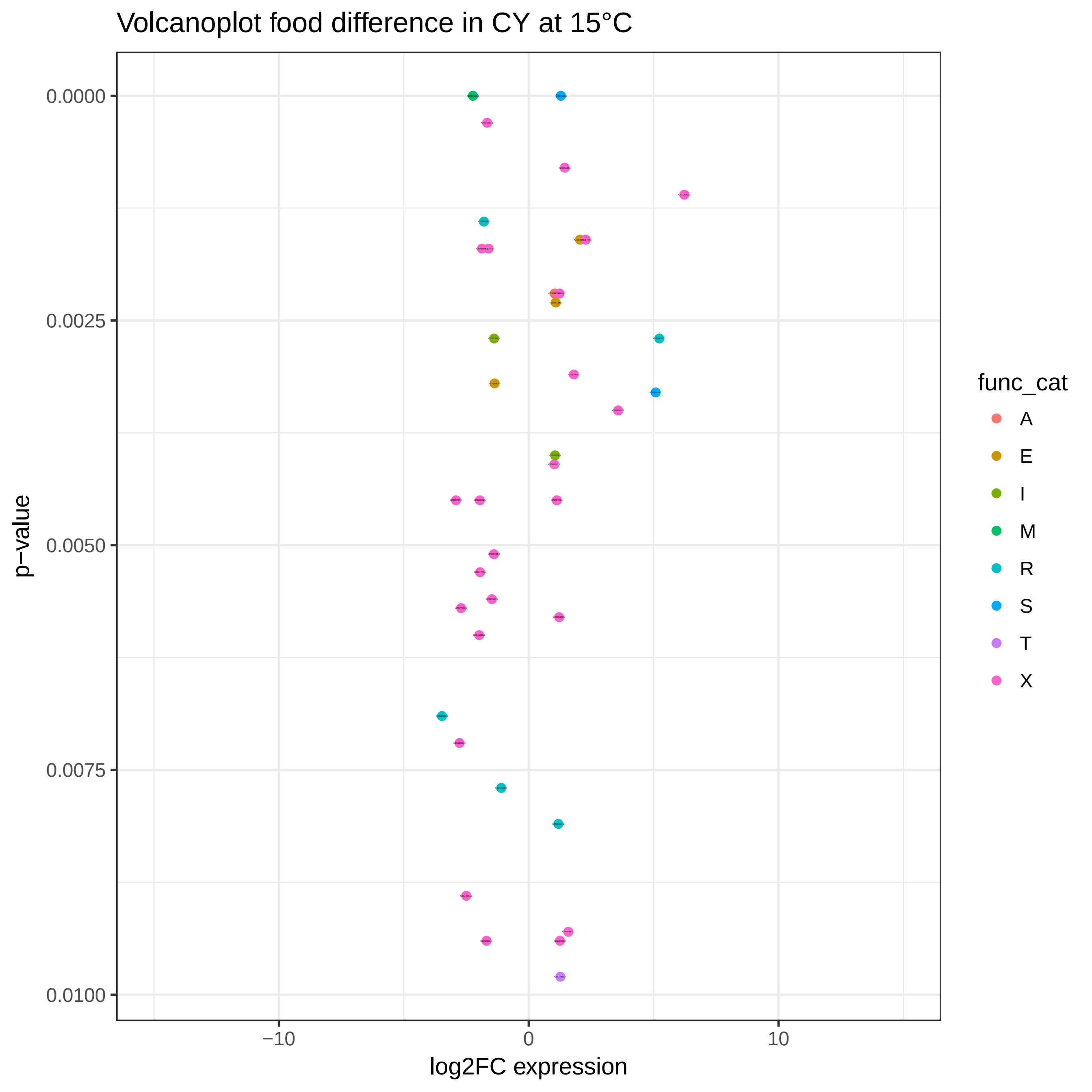

Volcanoplot food difference in CY at 15°C
p−value
0.0000
0.0025
0.0050
0.0075
0.0100
log2FC expression
−10
0
10
func_cat
A
E
I
M
R
S
T
X

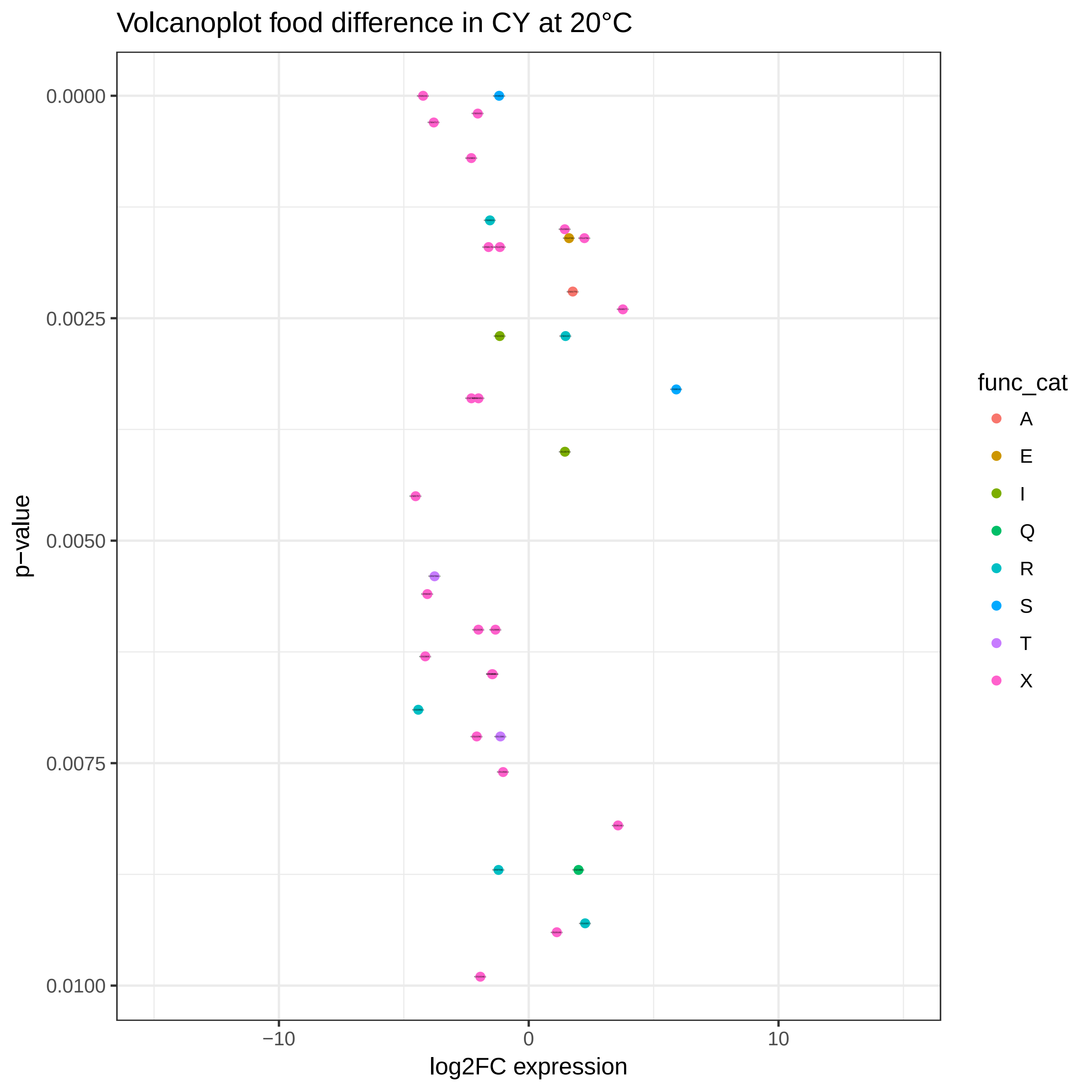

Volcanoplot food difference in CY at 20°C
p−value
0.0000
0.0025
0.0050
0.0075
0.0100
−10
0
10
log2FC expression
func_cat
A
E
I
Q
R
S
T
X

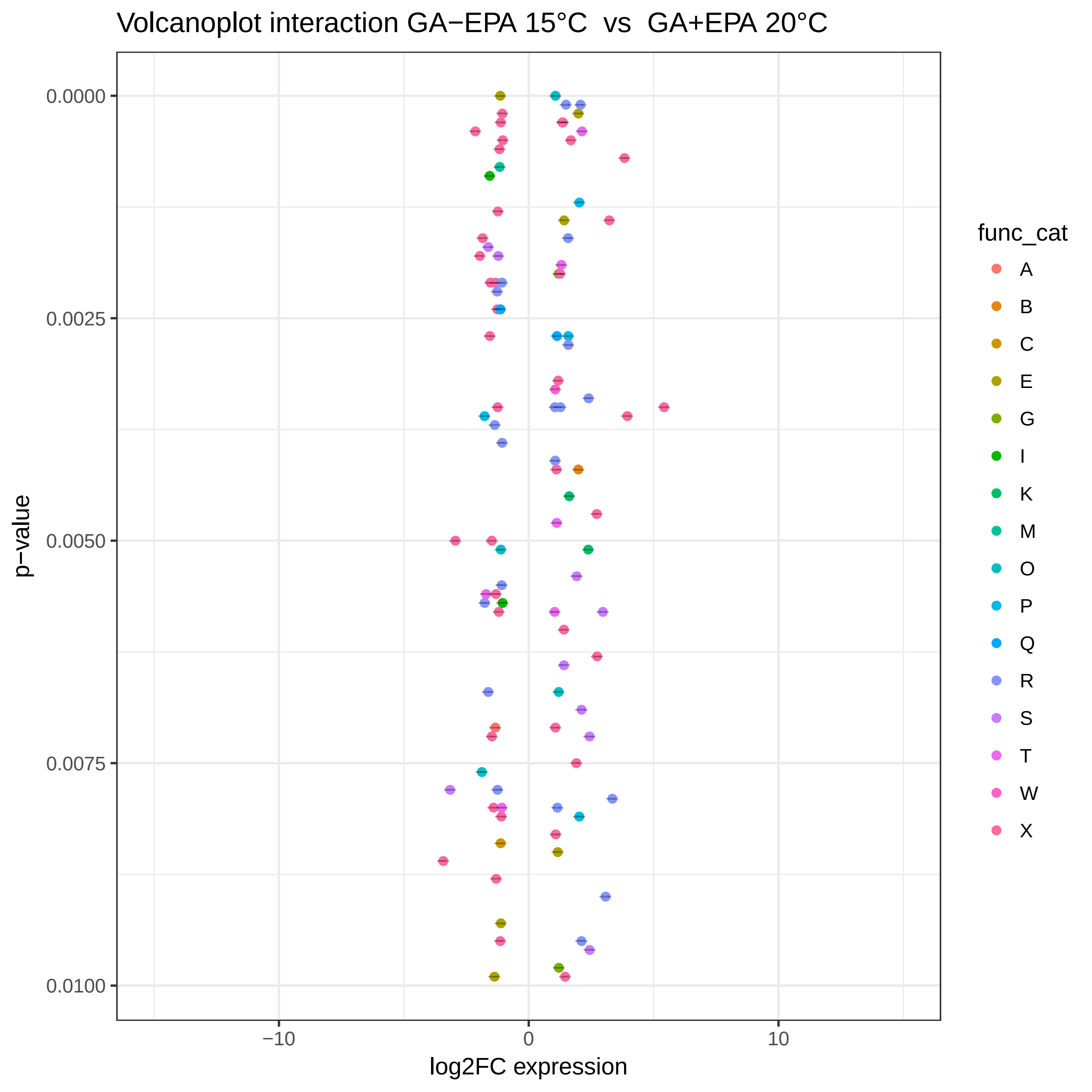
Volcanoplot interaction GA−EPA 15°C vs GA+EPA 20°C
p−value
0.0000
0.0025
0.0050
0.0075
0.0100
log2FC expression
−10
0
10
func_cat
A
B
C
E
G
I
K
M
O
P
Q
R
S
T
W
X

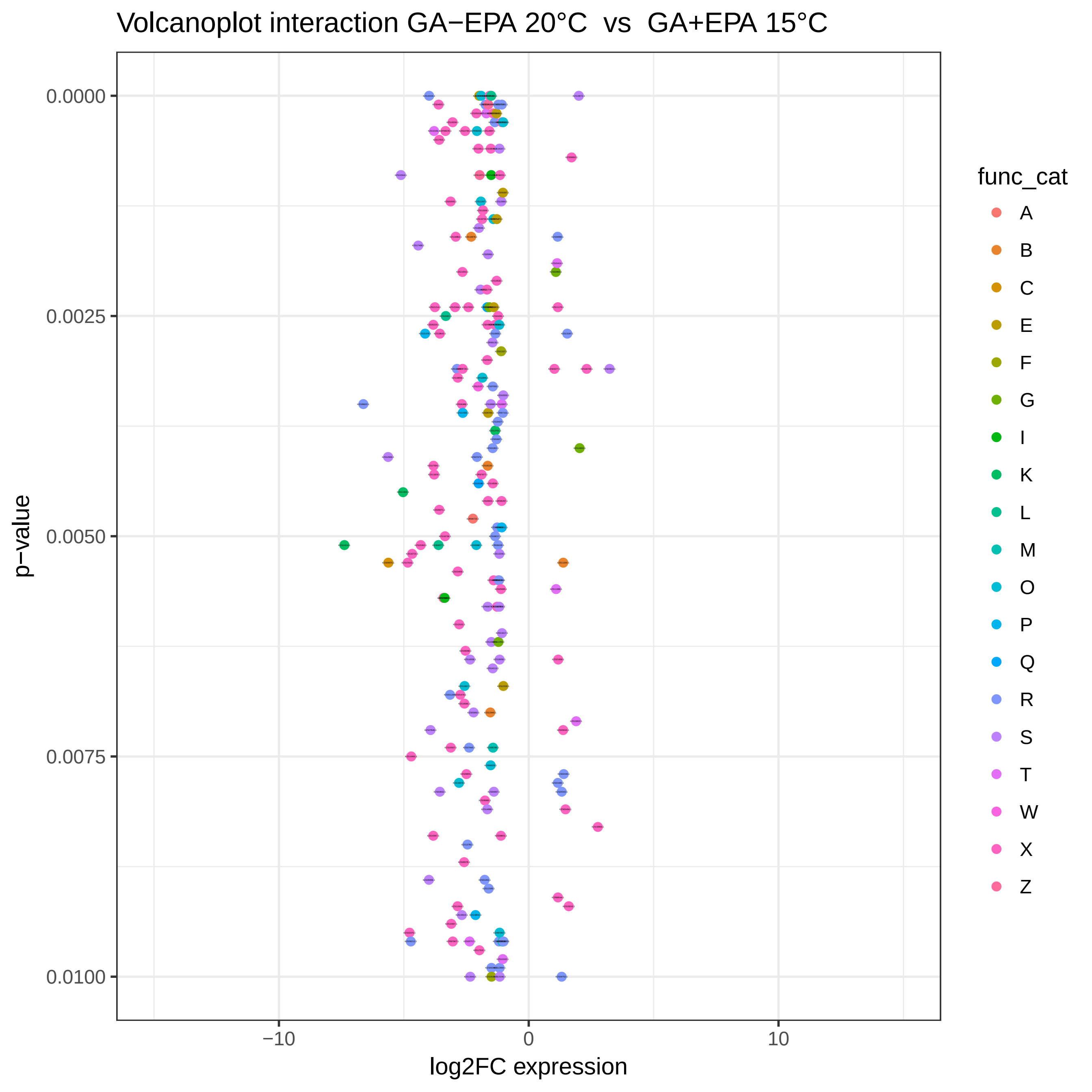
Volcanoplot interaction GA−EPA 20°C vs GA+EPA 15°C
p−value
0.0000
0.0025
0.0050
0.0075
0.0100
log2FC expression
−10
0
10
func_cat
A
B
C
E
F
G
I
K
L
M
O
P
Q
R
S
T
W
X
Z

Volcanoplot interaction CY−EPA 15°C vs CY+EPA 20°C

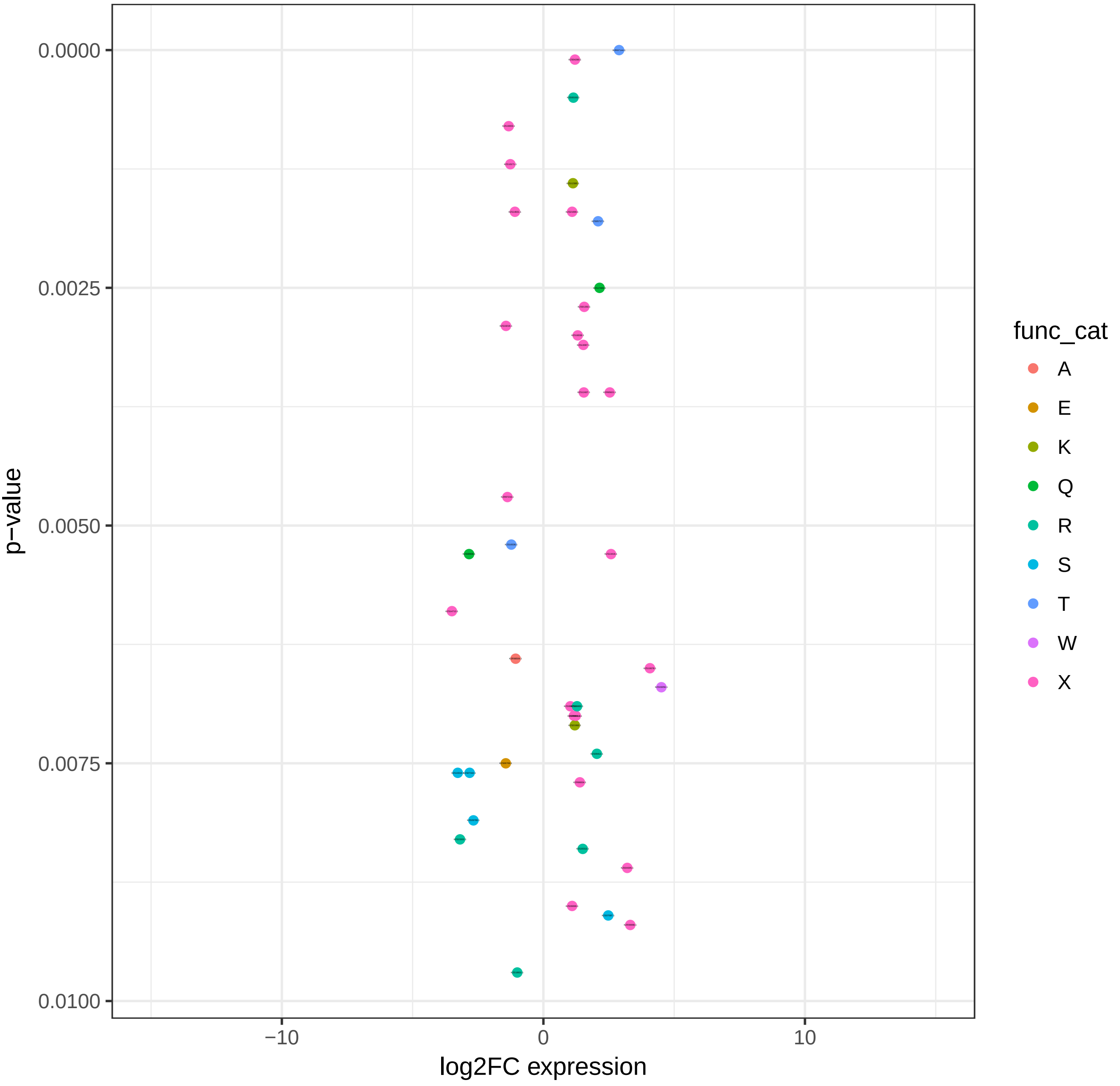

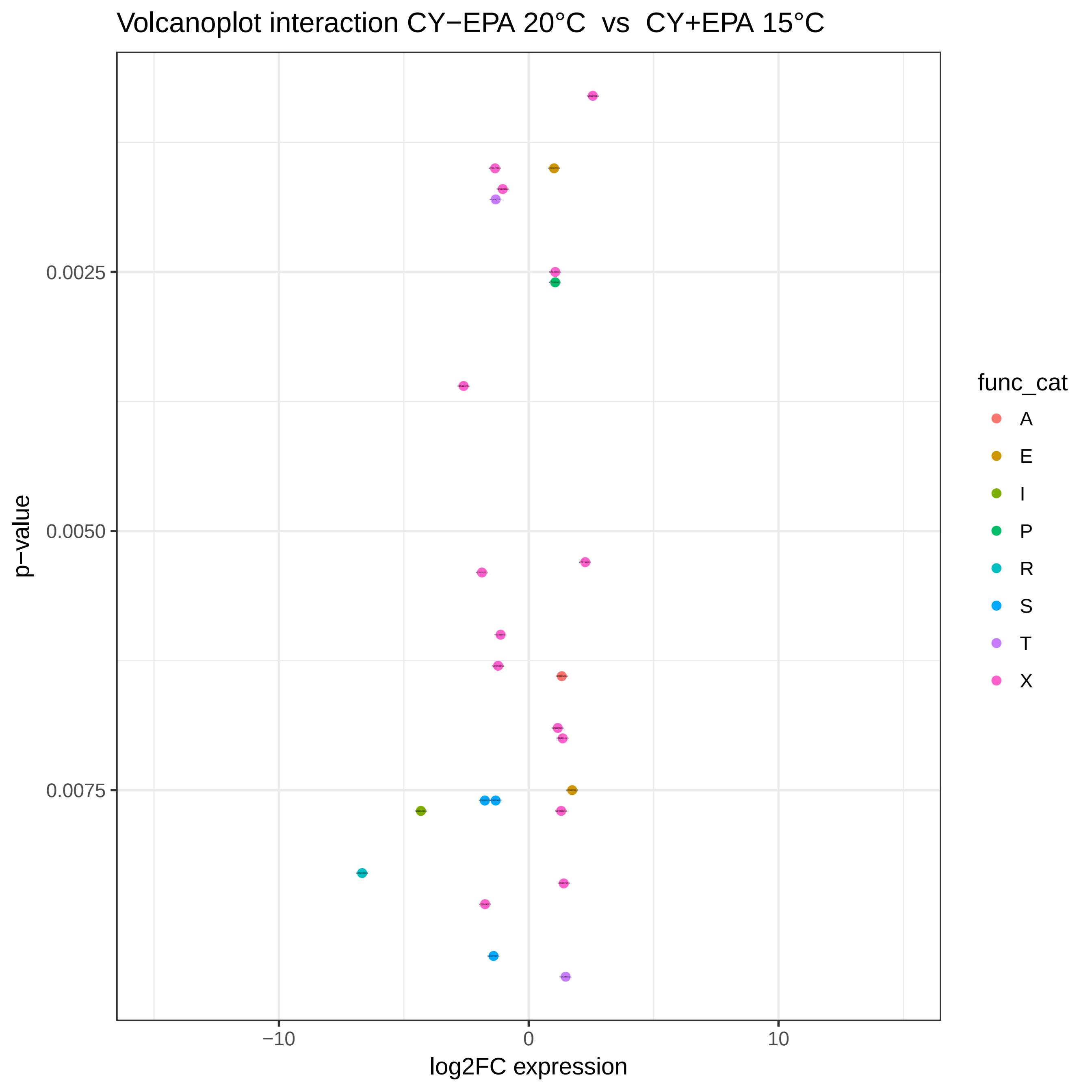
Volcanoplot interaction CY−EPA 20°C vs CY+EPA 15°C
p−value
0.0025
0.0050
0.0075
−10
0
10
log2FC expression
func_cat
A
E
I
P
R
S
T
X
